# Spatial Multi-Omics Integration with Mass Spectrometry Imaging: Decoding Metabolic Landscapes and Disease Mechanisms

**DOI:** 10.34133/csbj.0257

**Published:** 2026-11-12

**Authors:** Xiaorui Bu, Lufang Wang

**Affiliations:** Department of Obstetrics and Gynecology, Union Hospital, Tongji Medical College, Huazhong University of Science and Technology, Wuhan 430022, China.

## Abstract

Spatial multi-omics links molecular measurements to tissue architecture, but integrating spatial transcriptomics with mass spectrometry imaging (MSI) remains challenging because the modalities differ in sampling unit, spatial resolution, data structure, and molecular-identification certainty. This review synthesizes the enabling technologies and presents an evidence-calibrated workflow spanning tissue acquisition, spatial registration, modality-specific preprocessing, cross-modal annotation, mechanistic modeling, and orthogonal validation. We examine how MSI can complement single-cell and spatial transcriptomic or proteomic measurements in studies of tumor–host interfaces, metabolic dysfunction-associated steatotic liver disease, adipose-tissue organization, and decidualization at the maternal–fetal interface. Across these applications, spatial colocalization is most informative when annotation confidence is reported and key chemical identities are validated, registration error is quantified, and candidate cell–metabolite relationships are tested by isotope tracing or perturbation. We further discuss molecular ambiguity, resolution–sensitivity–throughput trade-offs, 3-dimensional reconstruction, interlaboratory reproducibility, and requirements for clinical translation. The central conclusion is that spatial multi-omics is best used to formulate anatomically explicit, falsifiable hypotheses rather than to infer causality from aligned maps alone.

## Introduction

Analysis of cellular heterogeneity is increasingly important for disease classification, treatment stratification, and the development of precision medicine. Single-cell genomic and epigenomic methods can resolve cell states that are obscured in bulk measurements, although their clinical value depends on appropriate sampling, validation, and interpretation [1]. In cancer, intratumoral heterogeneity contributes to disease evolution and therapeutic resistance, and longitudinal assessment of cellular composition can inform mechanistic studies and treatment design [2,3]. Single-cell technologies therefore provide a useful framework for identifying clinically relevant subpopulations and candidate therapeutic vulnerabilities [4].

Single-cell RNA sequencing (scRNA-seq) measures transcripts but does not directly capture proteins, lipids, or small-molecule metabolites. As reviewed by Liu *et al*. and by Buccitelli and Selbach, protein levels depend on transcription, translation, and mRNA and protein turnover. The relationship between mRNA and protein abundance therefore varies with the genes, biological conditions, and timescale examined. At single-cell resolution, Specht *et al*. used SCoPE2 proteomics and parallel scRNA-seq measurements from separate cells in the same monocyte-to-macrophage system to examine transcriptional and posttranscriptional regulation. Barry *et al*. [5] demonstrated temporal decoupling after hypoxic stress: Pseudotemporal alignment of separately measured transcriptomes and proteomes revealed an early transcriptional response followed by delayed protein changes. Related work on RNA–chromatin integration and single-cell lipidomics illustrates the distinct measurement properties and analytical requirements of additional molecular layers [6–10].

Multi-omics studies in environmental, plant, and proteome–metabolome research show that combining sequencing- and mass spectrometry (MS)-based measurements can link molecular regulation to metabolic phenotypes, but the strength of that link depends on experimental design and cross-modal validation [11–13]. Recent reviews have examined complementary aspects of spatial molecular mapping. Yang *et al*. [14] survey MS-based spatial omics, including technological and computational advances and biomedical applications. Geng *et al*. [15] systematically compare computational integration frameworks and fusion strategies, with datasets predominantly comprising transcriptomic–proteomic or transcriptomic–epigenomic measurements. The *npj Imaging* review by Zhang *et al*. [16] emphasizes mass spectrometry imaging (MSI) platforms, molecular sensitivity and specificity, and multimodal mapping, while Wheeler *et al*. [17] synthesize biomedical spatial metabolomics applications and analytical challenges.

This review focuses on how tissue MSI features associated with metabolites and lipids can be linked to cell states defined by spatial transcriptomics (ST) to formulate testable hypotheses about metabolic mechanisms. Single-cell transcriptomics and proteomics provide reference cell states and regulatory context. We connect computational method selection to the spatial and chemical evidence needed to support biological claims from MSI–ST data. An evidence-calibrated framework guides this assessment through molecular identity, spatial correspondence, cellular attribution, mechanistic plausibility, flux or transport validation, perturbational causality, and clinical utility. These checkpoints are claim-dependent requirements, not a mandatory linear sequence. Molecular identity, spatial correspondence, and cellular attribution can be established through partly independent experiments. A clinical prediction model may demonstrate utility for a defined decision without resolving the complete metabolic mechanism, provided that its analytical performance and clinical benefit are established. These requirements guide registration, resolution harmonization, multimodal representation learning, annotation-aware pathway analysis, and orthogonal or functional validation. Tumor, liver, adipose, and reproductive examples illustrate what current evidence supports and which experiments are needed to advance each interpretation.

## Foundational Technologies: Detection Strategies at Single-Cell Resolution

### Nucleic acid-based cellular atlas construction

#### Defining cell subpopulations and lineages with scRNA-seq

scRNA-seq profiles transcriptomes at cellular resolution and thereby reduces the averaging inherent in bulk RNA sequencing. Tang *et al*. [18] reported whole-transcriptome messenger RNA sequencing from a single mouse blastomere using complementary deoxyribonucleic acid amplification and next-generation sequencing. Subsequent high-throughput methods combined massively parallel profiling with unsupervised clustering to identify cell populations without predefined markers [19]. These approaches have enabled systematic analysis of cellular heterogeneity [20]. In cancer, they can distinguish malignant subclones and immune states and support inference of cell–cell communication within the tumor microenvironment (TME) [21,22]. Continued improvements in throughput, sensitivity, and analysis have expanded cell-type classification and discovery across complex tissues [23]. Nevertheless, transcript abundance does not fully determine protein activity or metabolic state; transcriptomic profiles must therefore be interpreted alongside proteomic and metabolomic measurements when the aim is to infer cellular function.

#### Capturing spatial heterogeneity of gene expression *in situ* with spatial transcriptomics

Tissue dissociation removes the spatial context of scRNA-seq data. ST addresses this limitation by measuring RNA abundance while retaining tissue coordinates, enabling gene-expression patterns to be related to histological architecture [24,25]. ST methods include imaging-based approaches, such as multiplexed error-robust fluorescence *in situ* hybridization (MERFISH) [26], and sequencing-based approaches that capture transcripts on spatially barcoded substrates, as illustrated by Slide-seq [27]. Early high-definition spatial transcriptomics (HDST) used arrays of approximately 2-μm barcoded features [28]. These methods connect gene-expression programs to tissue organization and local cell–microenvironment relationships [24,25].

Representative high-resolution platforms now span transcriptome-wide capture and targeted *in situ* imaging. Visium HD uses contiguous 2 × 2 μm barcoded squares and supports whole-transcriptome profiling, including a formalin-fixed, paraffin-embedded (FFPE)-compatible probe-based assay covering protein-coding genes. Stereo-seq uses DNA nanoball arrays with a 0.5-μm center-to-center pitch. Xenium Prime 5K and CosMx 6K instead localize individual transcripts by imaging and generate cell-level matrices after segmentation, with targeted panels of approximately 5,000 and 6,000 genes, respectively. A 2025 benchmark compared these 4 platforms in human tumors against matched scRNA-seq and adjacent CODEX measurements, evaluating sensitivity, specificity, transcript diffusion, segmentation, annotation, and spatial concordance. Its use of 8-μm bins for bin-level comparisons illustrates why native feature size and analytical resolution must be reported separately [29].

Other sequencing-based methods provide complementary sampling designs. Slide-seqV2 retains 10-μm beads while improving transcript capture sensitivity over Slide-seq. Seq-Scope uses RNA-capturing barcode clusters spaced approximately 0.5 to 0.8 μm apart. Slide-tags spatially barcodes individual nuclei before sequencing, combining whole-transcriptome single-nucleus profiles with spatial positioning reported at less than 10 μm in the mouse hippocampus. Barcode pitch, transcript localization, and cell or nucleus position thus describe different measurement properties. Effective biological resolution additionally depends on molecular capture, transcript diffusion, segmentation, aggregation and spatial sampling coverage. These distinctions determine the scale at which ST measurements can be paired with MSI [30–32].

### MS-based functional omics profiling

#### MSI: *In situ* mapping of metabolites and lipids

MSI maps ion signals directly across tissue sections without requiring analyte-specific labels. Depending on ionization method, mass analyzer, sample preparation, and spatial resolution, MSI can detect broad panels of ions associated with metabolites, lipids, peptides, or other molecular species while preserving their spatial distributions [33]. Matrix-assisted laser desorption/ionization MSI (MALDI-MSI) is widely used to visualize ion patterns assigned to metabolites, amino acids, and lipids in tissue [34]. Desorption electrospray ionization MSI (DESI-MSI), an ambient-ionization approach, can analyze minimally prepared tissue surfaces and has shown tissue-classification feasibility in clinical specimens [35]. MSI has also been used to quantify region-specific drug distributions in tumors, although rigorous interpretation requires attention to ion suppression and matrix effects [36].

MSI sensitivity and molecular coverage vary across analyte classes because matrix chemistry, ionization polarity, and tissue preparation affect extraction, desorption, and ionization differently. On-tissue derivatization can improve detection of poorly ionizing small molecules by introducing a charge or a readily ionized group. For example, Vicari *et al*. [37] used a reactive matrix that tags phenolic hydroxyl and primary amine groups to visualize neurotransmitter-related metabolites. The metabolic landscape observed by untargeted MSI therefore reflects both biological abundance and the chemical selectivity of the workflow. Reporting derivatization targets and class-specific coverage alongside matrix, polarity, and preparation conditions clarifies which molecular classes are represented in each dataset.

Throughout this review, we distinguish detected ion features, putatively annotated compounds, and structurally validated molecular identities. A detected ion feature is a signal defined by its mass/charge ratio (*m*/*z*), ionization polarity, and spatial intensity pattern, without an established chemical identity. Accurate mass, isotope patterns, and adduct evidence constrain formula-level candidates. A putative structural annotation additionally uses diagnostic fragmentation or spectral-library evidence at the structural level resolved; chemically plausible alternatives may remain. A structurally validated identity requires comparison with an authentic reference standard and complementary analytical evidence sufficient to discriminate the relevant alternatives at the structural level claimed. Established reporting schemes are provided by the Metabolomics Standards Initiative [38] and the framework of Schymanski *et al*. [39], although their level numbers refer to different criteria.

MSI platforms. The principal platforms differ in how ions are generated. MALDI uses laser irradiation of a matrix-coated surface to desorb and ionize analytes. Its molecular coverage and spatial fidelity depend on matrix chemistry and deposition [40]. DESI directs charged solvent droplets onto the tissue surface to extract and ionize analytes under ambient conditions without a conventional MALDI matrix. Solvent composition and spray geometry determine extraction behavior and the sampling footprint [41]. The ionization source governs ion generation, whereas the mass analyzer determines how those ions are measured. Time of flight (ToF), Orbitrap, and Fourier transform ion cyclotron resonance (FTICR) analyzers differ in acquisition speed, mass resolving power, and mass accuracy. These trade-offs guide platform selection for the ions and spatial scale of interest.

Secondary ion mass spectrometry (SIMS) uses a focused primary ion beam to sputter secondary ions from the surface. Molecular and fragment-ion coverage depends on beam type, dose, and instrument configuration. ToF-SIMS can connect ion and fragment profiles with cellular markers, as illustrated by scSpaMet, while OrbiSIMS combines SIMS sampling with high mass resolving power and MS/MS capability [42]. Because SIMS often produces fragment-rich spectra, structural and pathway assignments require evidence linking the detected fragments to their parent molecules.

From tissue to biological interpretation. An end-to-end MSI workflow progresses from a documented tissue specimen to coordinate-linked spectra, a processed pixel-by-ion-feature matrix, ion images, candidate molecular annotations, and biological hypotheses. The stages below describe the main inputs and outputs; their order depends on the platform and study design.

1. Tissue preparation. Preparation yields a mounted tissue section linked to an optical or histological reference image and records of specimen collection, stabilization, and anatomical origin. For labile metabolite and lipid studies, rapid stabilization, controlled storage, reproducible section thickness, and platform-compatible substrates are important. The choice between fresh-frozen and FFPE tissue determines the preparation procedure, while washing, retrieval, digestion, or derivatization is selected for the target analyte class. MALDI additionally requires controlled matrix deposition, whereas DESI and SIMS have their own surface and sampling requirements. Preparation conditions and linked same-section or serial-section histology should be documented to help interpret apparent differences caused by analyte loss or redistribution.
2. Acquisition and quality control. The prepared section is sampled at recorded *x*-*y* positions to produce spectra with spatial coordinates and acquisition metadata. The study should specify ionization source, mass analyzer, polarity, *m*/*z* range, mass calibration, resolving power, raster step, sampling footprint, and signal accumulation. Laser settings, DESI solvent and spray geometry, or SIMS beam and dose parameters should be reported as applicable. The raster step is the distance between acquisition positions; nominal pixel size describes the reconstructed sampling grid. The sampling footprint is the tissue area contributing to a spectrum. Effective biological resolution is the scale at which molecular differences can be assigned reliably to tissue structures or cells, accounting for the footprint, analyte delocalization, sensitivity, and registration or segmentation error. Increasing signal accumulation or mass resolving power can improve spectral quality at the cost of throughput. Blanks, off-tissue measurements, calibration checks, and suitable reference materials help distinguish background, drift, and biological signal.
3. Raw data and exchange formats. The raw dataset contains a spectrum at each sampled coordinate, together with specimen and instrument metadata. Spectra contain profile or centroid *m*/*z*–intensity pairs, which provide the starting point for feature extraction and molecular annotation. Vendor files can be retained alongside an interoperable imzML export, whose XML component describes spectra, coordinates, and metadata, while the linked.ibd file stores binary spectral arrays [43]. Conversion should preserve coordinate ordering, mass precision, intensity values, and provenance.
4. Spectral preprocessing and feature extraction. Raw spectra are converted into a quality-filtered pixel-by-ion-feature matrix with associated coordinates and quality-control flags. Depending on data representation, processing can include recalibration or mass alignment, baseline correction, noise filtering, justified smoothing, peak detection, and peak matching across pixels [44]. Signal-to-noise thresholds and mass tolerances determine which features are retained: A broad matching window can merge distinct ions, whereas an overly narrow window can split a drifting peak or increase missingness. Spatial masks should distinguish tissue, off-tissue controls, and failed measurements. Nondetection can reflect limited sensitivity or preprocessing thresholds as well as biological absence.
5. Normalization and ion-image reconstruction. Normalization improves the comparability of relative intensities across sampled locations; ion images are then reconstructed by mapping selected *m*/*z* intensities to their coordinates. Normalization may use total ion current, median-based scaling, or suitable internal standards, but its assumptions must be checked against the experimental design, including global and region-specific changes in signal composition. Comparison with appropriate standards or independent measurements should assess whether scaling suppresses a biological difference or creates a regional contrast. Deininger *et al*. [45] demonstrated that dominant localized signals can distort TIC-normalized protein images, highlighting the influence of tissue signal composition on normalization. Mass-extraction windows, intensity aggregation, spatial smoothing, and color scaling should be reported. Ion images show relative signal distributions, whereas absolute concentration measurements require analyte-specific calibration and validation.
6. Molecular annotation and validation. The annotation table links each feature’s mass, polarity, isotope, adduct, and spatial evidence to candidate formulas or compounds and the confidence of the assignment. It distinguishes detected ion features, putative annotations, and structurally validated identities. Evidence-based grouping of related adducts, isotopologues, and fragments reduces redundant assignments while preserving isotope information for tracer analysis. Formula-level false discovery rate (FDR) control can improve annotation [46], while key identities require standards and complementary evidence appropriate to the structural claim [38,47]. Unresolved alternatives should be retained in the annotation output. A practical workflow for matching MSI features to LC-MS/MS results and validating selected regions by laser microdissection is described in the *In situ* validation and perturbation testing section.
7. Statistical analysis and biological interpretation. Ion images, the processed matrix, and the annotation table can support region comparisons, spatial segmentation, classification, or cross-omics association [44]. The output is a set of spatial signatures and hypotheses with effect estimates and uncertainty. Because pixels from one specimen share biological and spatial context, analysis should use independent specimens as biological replicates and account for spatial autocorrelation, multiple testing, and acquisition batches. Registration links MSI features to histology and transcriptomic or proteomic sampling units at the resolution supported by the measurements. Pathway analysis should account for candidate identities and related ion forms, with ambiguity-aware enrichment used to test the effect of alternative assignments.

Conventional MSI maps the spatial distribution of relative ion signals, which reflect molecular abundance as well as ionization and matrix effects. It does not directly measure metabolic flux, reaction rates, metabolite turnover, uptake, secretion, or pathway directionality. An increased molecular pool can arise from greater production or delivery, reduced consumption or export, or altered cellular composition. Flux estimation combines isotope-labeling or kinetic measurements with models, using a defined labeling design, sampling interval, and set of assumptions. Stable-isotope MSI and spatial isotopologue modeling provide a route to such estimates, while perturbation experiments test the functional dependence of the proposed mechanism.

#### Single-cell proteomics: Ultrasensitive MS-based quantification

Protein abundance and activity depend on regulation beyond transcript abundance. Unlike nucleic acids, proteins cannot be amplified before analysis, making deep single-cell quantification technically demanding. MS-based single-cell proteomics (SCP) has advanced through improvements in low-input sample preparation, automation, ion handling, acquisition, and data analysis. Automated preparation workflows can improve reproducibility and scalability for low-input proteomic samples [48]. Faster, more sensitive instruments and efficient ion sampling have increased proteome depth and throughput. Orbitrap Astral workflows use rapid, sensitive MS/MS acquisition with narrow-window data-independent acquisition (DIA) to deepen single-cell proteome coverage. timsTOF SCP/Ultra workflows combine trapped ion mobility separation with parallel accumulation-serial fragmentation in DIA mode (diaPASEF), enabling sensitive profiling at very low input with optimized LC-MS throughput. In these workflows, shorter LC gradients increase throughput but can reduce proteome depth [49,50]. Attainable coverage also depends on low-loss preparation, chromatographic performance, cell type, and analysis settings. High-field asymmetric waveform ion mobility spectrometry (FAIMS)-assisted DIA is a complementary acquisition approach [51].

Computational advances support reliable identification and quantification from sparse single-cell measurements. In SCoPE2, DO-MS optimizes data acquisition, while DART-ID uses Bayesian retention-time alignment to refine peptide-identification confidence with FDR control. DIA-NN combines neural-network scoring with interference correction for DIA identification and quantification. Downstream analysis must also account for acquisition batches, cell quality, shared peptides, and missing measurements. scplainer fits peptide-level linear models with biological and technical covariates to estimate differential abundance and explore major sources of variation without imputing missing values. This preserves the distinction between observed intensities and inferred effects. When biological groups are confounded with acquisition batches, experimental balancing and independent replication remain necessary to distinguish those effects [10,52,53].

Measurements from the same biological system, as in SCoPE2, allow direct comparison of transcriptomic and proteomic variation. Trajectory alignment additionally addresses asynchronous molecular responses: Barry *et al*. [5] resolved a lag between transcriptional and protein-level responses to hypoxia. In spatial multi-omics, SCP can therefore supply measured enzyme abundance and protein-response programs to complement transcript-derived constraints, with coverage, peptide-to-protein mapping, and uncertainty carried into downstream models [10].

## Strategy and Workflow for Integrated Spatial Multi-Omics Analysis

### Methodological and analytical workflow

The workflow translates this evidence framework into tissue acquisition, spatial registration, modality-specific preprocessing, cross-modal annotation, mechanistic modeling, and orthogonal validation (Fig. 1). MSI contributes coordinate-linked feature matrices and annotation tables to this workflow. Table 1 compares representative computational strategies by analytical objective, required inputs, assumptions, and validation requirements, and distinguishes direct paired MSI–ST evaluations from evidence in related modalities and prospective MSI–ST extensions. Registration links tissue coordinates, deconvolution estimates cellular mixtures, and MSI annotation ranks chemical candidates within one molecular layer. Joint representation learning combines molecular layers, whereas association analysis tests relationships between matched measurements. Each task has distinct requirements for spatial scale, batch effects, and missing data.

**Fig. 1.**
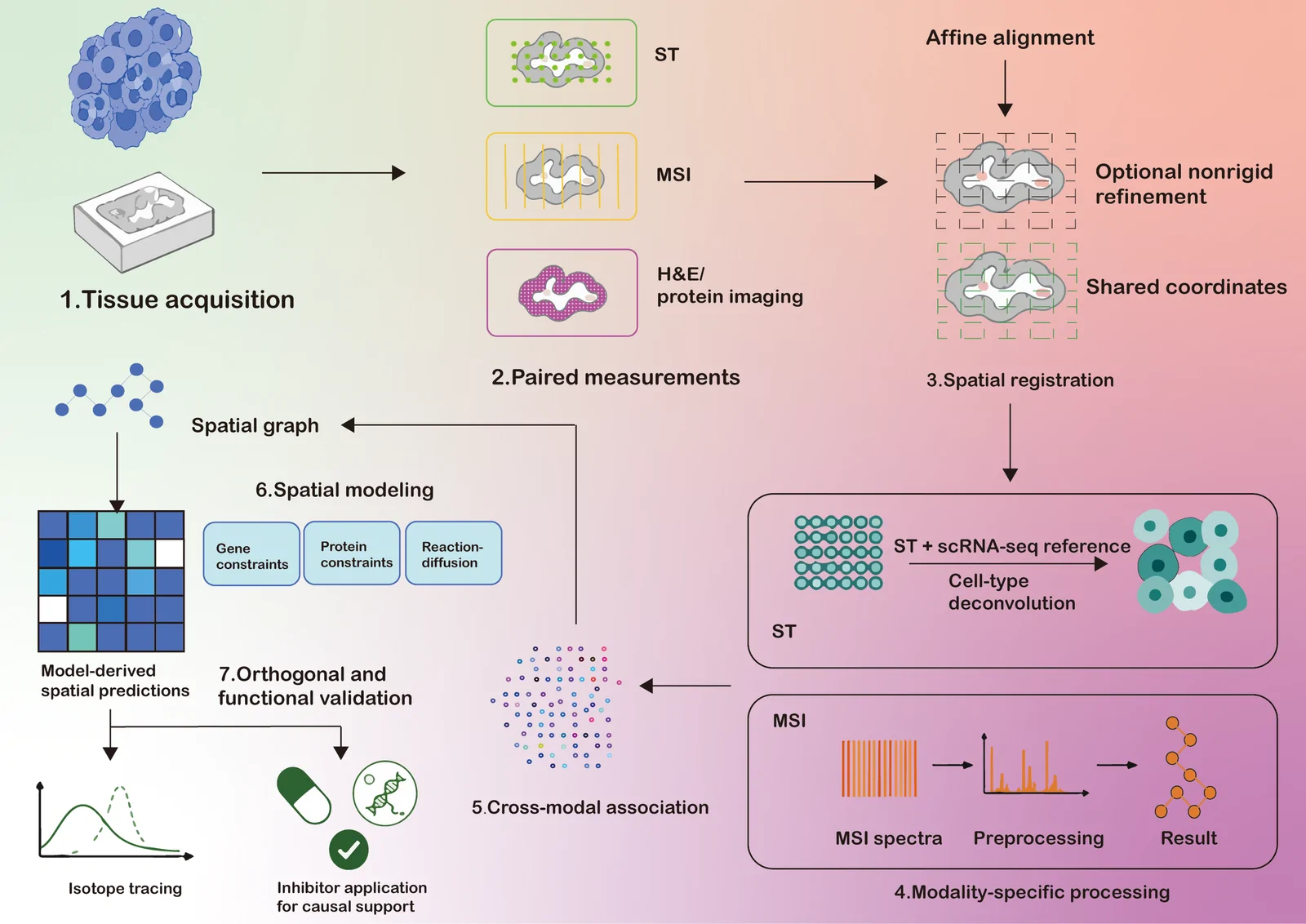
Workflow for integrating spatial transcriptomics and MSI. Tissue acquisition (1) is followed by paired ST, MSI, and histological or protein measurements (2). Same-section acquisition reduces inter-section correspondence uncertainty, while segmentation, unequal sampling footprints, and compatibility between sequential assays still require validation. Affine alignment and optional nonrigid refinement bring the measurements into shared coordinates (3). Modality-specific processing (4) combines cell annotation or reference-based deconvolution of ST data with preprocessing and molecular annotation of MSI spectra. The MSI “Result” represents ion features and their candidate annotations. Cross-modal association (5) links the molecular features to cell types and states. These associations inform spatial graphs and models constrained by gene expression, protein measurements, and reaction–diffusion relationships (6). The model-derived spatial predictions guide isotope tracing and perturbation experiments (7). Isotope tracing informs flux estimates, while inhibitor or genetic perturbations test the functional contribution of proposed pathways. The numbered stages describe an analytical workflow; the evidence required depends on the intended claim. H&E, hematoxylin and eosin; MSI, mass spectrometry imaging; scRNA-seq, single-cell RNA sequencing; ST, spatial transcriptomics.

**Table 1.** Comparative selection of computational strategies for spatial multi-omics integration. Evidence labels describe the specific task demonstrated in the cited studies. “Paired” includes same-section and registered serial-section measurements, whose spatial uncertainties differ. “Related-modality component” denotes evaluation in transcriptomic or other molecular data; direct ion-to-cell attribution is not established by that evaluation. “Prospective” denotes the MSI–ST extension discussed here for which the cited method study does not provide direct validation. Feature alignment connects molecular representations and is distinct from coordinate registration. Batch mixing, smooth maps, and reconstruction accuracy should be assessed alongside preservation of biology.

Strategy and evidence	Inputs and main assumptions	Resolution, batch effects, and missing modalities	Validation and principal failure mode
MIIT/GreedyFHist Registration and footprint fusion Paired MSI–ST [54]	Corresponding serial-section histology, ST and MSI coordinates; identifiable shared anatomy. Shared genes or ion identities are unnecessary.	Aggregates transformed measurements to target footprints. Molecular batch correction is separate; does not reconstruct an absent modality.	Held-out landmark displacement and region overlap (e.g., Dice coefficient). Tissue loss, noncorresponding anatomy, or deformation can produce false matches.
STalign Image-based registration Paired application [60,138]	Cell-density or histology representations and shared anatomy; paired molecular features are unnecessary. Applied to adjacent-section liver MSI–ST.	Transforms coordinates; footprint aggregation and molecular batch correction remain separate. Does not infer an absent molecular layer.	Independent landmark displacement and mask overlap. Anatomical agreement can conceal molecular differences or over-warping.
PASTE/PASTE2 Optimal-transport alignment Related modalities; MSI–ST extension prospective [61,62]	ST slices with shared genes and spatial geometry; PASTE2 allows partial overlap. MSI–ST needs a justified cross-modal feature cost.	Allows unequal observation counts; does not establish cell identity or correct MSI batch effects. Missing overlap differs from a missing modality.	Landmark displacement, overlap recovery, and sensitivity to cost weighting. Incorrect feature links or overlap assumptions distort correspondence.
SpatialMETA Joint representation Paired MSI–ST [67]	ST counts, MSI intensities, coordinates, and sample labels; assumes useful correspondence after alignment and reassignment.	Reconciles sampling grids; batch-conditioned decoding supports cross-sample integration. Reconstruction of observed layers does not validate prediction of an absent modality.	Held-out molecular reconstruction error, domain agreement (e.g., adjusted Rand index), and biological conservation alongside batch mixing. Overcorrection or reassignment can obscure true differences.
SpatialGlue/MultiGATE Joint domain discovery Paired domain evaluations [57,67,90]	Matrices at corresponding locations and spatial graphs; MultiGATE adds cross-feature relationships. MSI–ST tests include third-party comparisons for SpatialGlue.	Requires prior resolution matching. Cross-sample batch handling needs separate evaluation; paired-input tests do not establish missing-modality recovery.	Domain agreement with independent annotations (e.g., adjusted Rand index) and boundary recovery. Smoothing or modality dominance can create coherent but misleading domains; attention is not causality.
cell2location/RCTD Cell-mixture deconvolution Related-modality component [80,84]	Mixed-cell ST counts and a cell-type-labeled scRNA-seq reference sharing genes; relevant populations must be represented. No paired MSI input is required.	Models technical or platform variation in RNA. MSI footprint matching is separate; estimates composition, not a missing metabolite layer.	Independent cell counts or markers; abundance error and sensitivity to reference omissions. Similar states or absent populations can bias mixtures.
Seurat v5 bridge Cross-modal feature alignment MSI–ST extension prospective [66]	Unpaired query modalities plus a measured paired multi-omic reference representing relevant states. MSI–ST would require a transcript–ion bridge.	Maps unpaired modalities through the reference; spatial registration and MSI-specific batch validation remain separate. An RNA–protein bridge cannot supply an unmeasured MSI layer.	Held-out paired targets and independent annotations. Reference mismatch or unsupported gene–ion links can drive incorrect transfer.
MANTIS Spatial association testing Paired MSI–ST; preprint [93]	Already aligned ST–MSI profiles, coordinates, and optional cell-type or domain annotations; relevant confounders must be represented.	Requires prior footprint matching and appropriate batch handling. Neither registration nor absent-modality reconstruction is its task.	Autocorrelation-preserving null calibration, multiple-testing control, and independent-specimen replication. Residual confounding or misregistration can mimic an association.

MSI, mass spectrometry imaging; ST, spatial transcriptomics; MIIT, Multi-Omics Imaging Integration Toolset; RCTD, robust cell type decomposition

Statistical design should follow the hierarchy of patients, sections, regions, and pixels or spots. Thousands of measurements from a few patients provide detailed spatial sampling but can produce pseudoreplication if treated as independent biological replicates. For patient-level inference, all measurements from each patient should remain in the same training, validation, or test partition (Table 4). Group comparisons can use patient-level summaries of prespecified regions or hierarchical models that account for measurements nested within patients and, where appropriate, residual spatial correlation. Confidence intervals can be estimated by resampling patients, while between-patient group-label permutations should preserve within-patient measurements and any pairing or study blocks. These choices ensure that uncertainty reflects the number of independent patients rather than the number of sampled pixels.

MSI preprocessing before integration: Cross-modal feature selection begins with a documented pixel-by-ion-feature matrix. Depending on spectral representation and acquisition mode, mass recalibration, noise and baseline treatment, peak detection, and peak alignment or *m*/*z* binning define the matrix columns. Tissue masks, off-tissue measurements, and matrix blanks guide background-feature filtering. Isotope and adduct relationships should be recorded to limit redundant molecular assignments while retaining isotopologue signals for tracing. Normalization should account for tissue composition and be checked for distortions associated with ion suppression or dominant ions. Quality control (QC) measurements and acquisition metadata then support assessment of drift and batch effects, with any correction evaluated for preservation of biological contrasts. Comparing retained features and regional patterns across reasonable preprocessing settings shows how these choices affect downstream associations. The resulting matrix should retain coordinates, QC flags, feature-group annotations, and processing parameters so that integration results can be traced to the measured spectra.

Molecular-identification confidence: Each MSI feature can be assigned a qualitative confidence category. Unassigned ion features retain their measured *m*/*z* without a molecular name. Accurate-mass/formula-level annotations nominate candidates using mass, isotope, and adduct evidence. Putative structural annotations add diagnostic MS/MS or spectral-library support at the structural detail resolved. Authentic-standard-supported identifications require agreement in at least 2 independent analytical properties measured against the standard under matched conditions. Orthogonal LC-MS/MS, retention-time comparison under the same chromatographic method, or ion-mobility evidence can further resolve alternatives, with each measurement’s specimen or region provenance recorded. Evidence for identity in an extract should be recorded separately from evidence assigning that identity to an MSI image. Retaining these categories, residual candidates, and supported structural detail allows pathway conclusions to be tested across alternative annotations [38,39].

#### Spatial registration and data fusion

Integrating ST with MSI requires accounting for differences in spatial resolution, data structure, and tissue morphology. ST measurements range from mixed-cell capture spots to subcellular transcript coordinates and segmented cell or nucleus profiles, whereas MSI records spectra from platform-dependent sampling footprints. Either modality may therefore provide the coarser spatial layer. The platforms also differ in tissue preparation and sampling geometry. Because ST and MSI are often acquired from adjacent or serial sections, stretching, tearing, compression and local warping can displace regions that correspond biologically [54]. Wess *et al*. [54] showed that ST and MSI data from serial prostate-tissue sections separated by up to 100 μm could be integrated computationally. Registration becomes more difficult as section distance, tissue heterogeneity, and damage increase.

These differences make image registration a prerequisite for reliable cross-modal analysis [55]. Local tissue deformation generally requires nonrigid, rather than purely affine, transformation models [56]. In the Multi-Omics Imaging Integration Toolset (MIIT), Wess *et al*. [54] combined affine and nonrigid registration using GreedyFHist to align stained serial sections and integrate ST with MSI. More broadly, spatial multi-omics analysis depends on maintaining consistent coordinate transformations across modalities [57]. Same-section workflows, such as the transcriptome–metabolome approach reported by Vicari *et al*. [37], reduce inter-section mismatch while retaining uncertainty from cell segmentation, sampling footprints, and assay compatibility. A practical registration pipeline should therefore include global initialization, local deformation correction, transformation of all molecular layers into a shared coordinate system, and quantitative assessment of registration error.

Specimen pairing and same-section acquisition: Same-section ST–MSI links measurements to a shared tissue plane and supports direct local correspondence while retaining the need to align images and reconcile sampling footprints. Its molecular coverage depends on preserving compatibility between sequential assays. Serial sections allow each assay to use its preferred substrate and preparation, although local 3-dimensional heterogeneity and deformation usually limit correspondence to regions rather than identical cells. Cross-specimen integration offers protocol independence and broader cohort coverage, with biological variability added to platform differences. It supports testing recurring cell-state or regional relationships across samples, whereas local molecular correspondence is assessed in paired material.

Assay order should be selected and validated for the target analytes and ST platform. MALDI-MSI before ST can capture metabolite distributions before ST-specific washes or permeabilization. Matrix deposition/removal, solvent exposure, and laser sampling then need to be evaluated for their effects on RNA integrity and recovery. Performing ST first can alter subsequent MSI signals through washing, staining, analyte delocalization, or tissue consumption. Compatible substrates and matched single-modality controls should therefore be used to assess RNA recovery, ion coverage, morphology, and spatial redistribution after each step. Published same-section studies illustrate how acquisition protocols can be designed jointly to improve correspondence [37,58].

After image alignment, measurements are matched according to their sampling footprints. When ST resolves cells or transcripts more finely than MSI, the spatial scale of joint analysis is limited by MSI. A practical approach is to aggregate ST counts or cell-type abundances across each MSI footprint using spatial overlap and segmentation masks, yielding a representation of the cellular mixture associated with that spectrum. When ST is coarser than MSI, MSI features can instead be aggregated within the ST sampling units for joint analysis. Transfer to finer MSI locations can then be formulated as probabilistic prediction, with uncertainty reflecting the unobserved within-spot composition. The common analysis scale should account for both modalities’ effective resolution and registration error. Retaining native coordinates and matrices alongside the aggregated representation makes these choices explicit and permits sensitivity analyses across spatial scales. Upsampling refines the computational grid without adding experimentally measured molecular detail.

Nonrigid and optimal-transport alignment: Comparative evaluations of ST methods distinguish slice alignment from data integration and assess their effects on downstream analyses [59]. STalign uses affine initialization and large-deformation diffeomorphic metric mapping (LDDMM) to align cell-density or histology images [60]. It accommodates deformation without shared molecular features, although similar anatomy can conceal different molecular states. PASTE combines shared gene-expression similarity with spatial distances through fused Gromov–Wasserstein optimal transport and can construct a low-rank consensus slice [61]. PASTE2 adds partial-overlap alignment [62]. These transport methods support unequal observation counts, but applying their molecular cost to ST–MSI requires validated cross-modal links or a paired reference. The feature–geometry balance and overlap estimate determine the favored correspondences (Fig. 2).

**Fig. 2.**
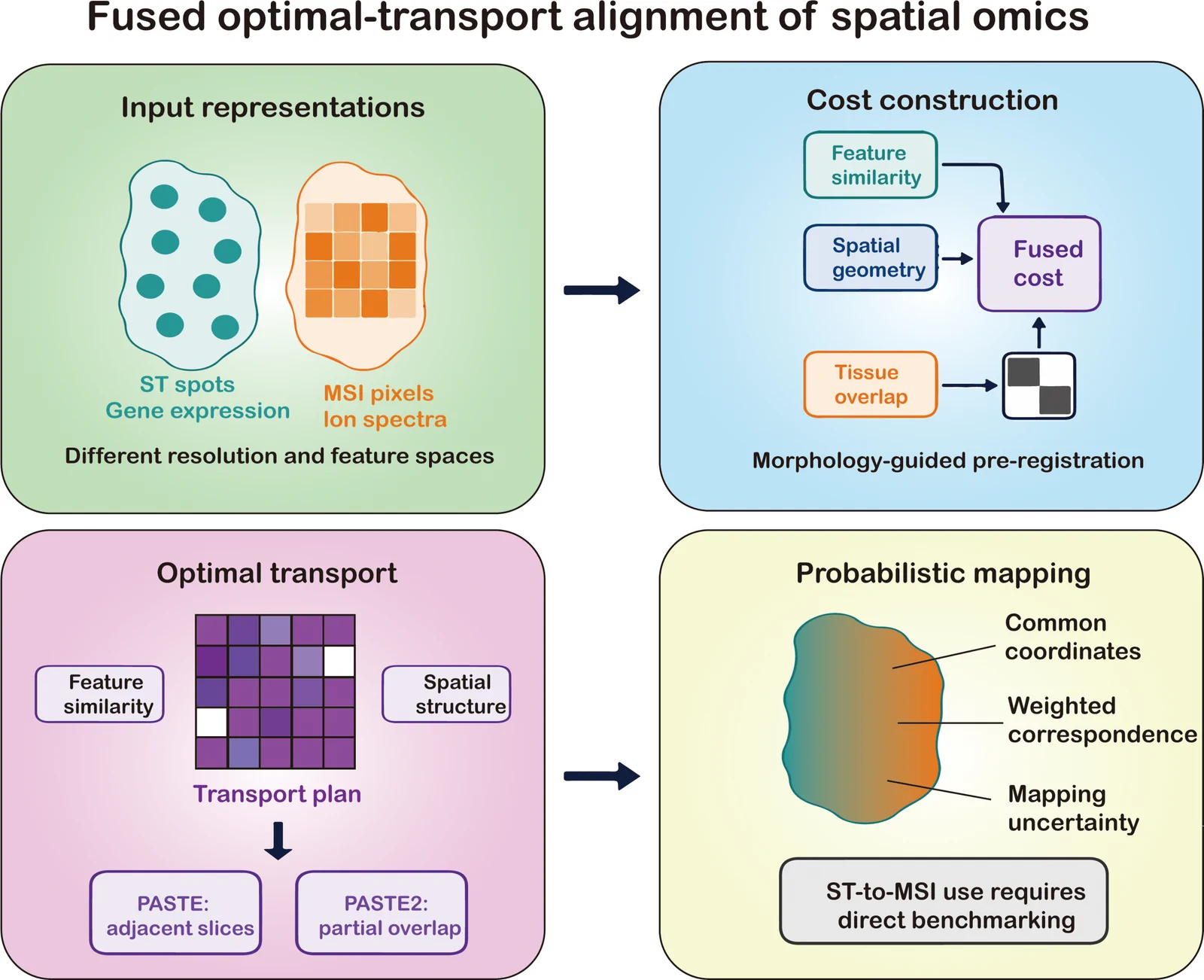
Conceptual fused optimal-transport alignment of spatial-omics measurements. Spatial-transcriptomic spots and MSI pixels differ in resolution and feature space. After morphology-guided pre-registration, a fused cost can combine cross-modal feature similarity, within-modality spatial geometry, and tissue overlap. Optimization produces a transport plan that represents weighted, probabilistic correspondence rather than one-to-one identity. PASTE illustrates alignment of adjacent spatial-transcriptomic sections, whereas PASTE2 accommodates partial overlap. Their direct use for ST–MSI alignment requires modality-specific benchmarking, uncertainty reporting, and validation against anatomical landmarks. MSI, mass spectrometry imaging; PASTE, probabilistic alignment and integration of spatial transcriptomics; ST, spatial transcriptomics.

Cross-modal interpolation and joint representation learning: SpaGCN combines ST expression, coordinates, and histology for spatial-domain detection [63]. Neighborhood smoothing favors coherent domains and makes boundary recovery sensitive to graph scale. ConGcR and ConGaR combine gene expression, spatial coordinates, and histology through contrastive learning [64], while GRAS4T combines histology-informed graph contrastive learning with subspace analysis for spatial-domain identification [65]. Seurat v5 bridge integration uses dictionary learning with a separate paired multi-omic reference [66]. It links unpaired datasets when the bridge represents the relevant states and modalities. An ST–MSI application therefore needs a bridge with measured transcript–ion relationships; an RNA–protein or RNA–chromatin reference does not supply these links.

SpatialMETA directly integrates ST and MSI through alignment, spatial reassignment, and a conditional variational autoencoder with separate reconstruction distributions for transcript counts and metabolite intensities. Batch-conditioned decoders and a distribution-matching loss support cross-sample integration. Its evaluations include kidney cancer, glioblastoma, and mouse brain data. The resulting niches depend on spatial reassignment and preprocessing, as well as on how well batch correction preserves biological variation [67].

Graph learning combines spatial neighborhoods with modality-specific features [57,68,69], complementing geometry-based fusion [37,54,70]. Coordinate matching, joint embedding, and gene–ion association address distinct tasks, each with its own outputs and sources of error [14,71]. Native sampling footprints require reconciliation before joint analysis. Partial tissue overlap, missing genes within a measured layer, and an entirely absent modality also require different treatment. Prediction of an unmeasured layer should be tested by masking observed data or using independent paired samples, with predictions identified separately from measurements.

Validation should distinguish 3 objectives. Registration accuracy is assessed using landmarks, fiducials, or anatomical masks excluded from fitting and tuning, reporting displacement error or region overlap. Predictive performance is assessed against held-out molecular measurements using spatially blocked or whole-specimen holdouts, with feature selection and model tuning confined to training data. Biological validity requires reproducible cross-modal associations after accounting for anatomical region, cellular composition, patient or batch effects, and residual spatial dependence. Accurate reconstruction can arise from shared morphology or spatial smoothness, so comparison with morphology-only or spatial-only baselines helps establish what molecular integration adds. Null models should preserve the relevant spatial autocorrelation, tissue mask, and experimental grouping while disrupting the relationship being tested. Block-based rearrangements require checks that block size, and tissue boundaries preserve that structure, and unrestricted coordinate shuffling can serve as a stress test but is insufficient on its own for calibrating statistical significance tests. Registration perturbations should reflect measured alignment errors, with changes in association direction and effect size reported. Repeated measurements across sections, runs, or platforms assess technical reproducibility; replication in independent patients, tissues, and disease settings tests biological generalizability within those settings.

Unified spatial data representation: Following registration and resampling, ST expression matrices and MSI ion-intensity matrices can be linked to a common coordinate system while retaining their native resolutions. SpatialData provides a standardized representation for raster images, point data, shapes, labels, transformations, and annotation tables across spatial-omics technologies [15,72]. A unified object can then store transcriptomic measurements and MSI ion features, together with candidate annotations, their supporting evidence, histology, segmentation, and spatial transformations. Additional modalities may be included when they are measured experimentally: Spatially resolved transcript amplicon readout mapping (STARmap) PLUS jointly profiles RNA and selected proteins [73], whereas microfluidic indexing-based spatial assay for transposase-accessible chromatin and RNA sequencing (MISAR-seq) combines spatial gene-expression and chromatin-accessibility measurements [74]. Recording the identity and provenance of each measured layer supports downstream annotation, niche analysis, and validation of cross-modal correspondence.

#### Cross-modal annotation and functional niche definition

Once spatial coordinates and molecular layers have been aligned, the next task is to translate feature matrices into cellular and tissue-level annotations. Spatial-domain detection and cell-type deconvolution provide complementary descriptions of tissue heterogeneity [75]. The appropriate operation depends on the ST measurement unit. Mixed-cell spots or analysis bins require estimation of cell-type composition [76], whereas segmented cell-level measurements require cell annotation and quality control of transcript assignment. MSI pixels may contain signals from more than one cell or extracellular compartment [58], including when the paired ST data resolve individual cells. Cross-modal annotation therefore relates cellular information to MSI at the measured sampling scale, with uncertainty estimates and independent validation for the resulting niche assignments [77,78]. The workflow comprises 2 related steps.

##### Single-cell reference-guided spatial deconvolution

For mixed-cell ST spots or bins, a matched scRNA-seq reference supports estimation of cellular composition [79]. cell2location models cell-type abundance and technical variation hierarchically [79], whereas robust cell type decomposition (RCTD) fits reference-derived mixtures with gene-specific platform correction [79,80]. Both depend on reference coverage, so omitted populations or closely related states can bias assignments. Histology can provide additional constraints: SegDecon uses image-derived nuclei counts to calibrate cell-abundance priors in a modified cell2location model [81]. SpatialScope uses reference-trained generative modeling to reconstruct cell-level profiles and predict unmeasured RNA genes [82], yielding more detailed expression profiles that depend more strongly on the reference. Benchmarks support cell2location and RCTD in several settings, with rankings dependent on tissue, reference quality, and metric [79,83,84]. These methods infer composition or expression within measured units, while MSI footprint matching remains a separate step. Segmented ST instead requires cell annotation and transcript-assignment quality control. Relevant annotated states may include exhausted cluster of differentiation 8 (CD8)-positive T cells, M2-like tumor-associated macrophages (TAMs), and cancer-associated fibroblasts (CAFs).

##### Cross-modal cell-identity transfer to MSI pixels

Unlike transcriptomic data, MSI records *m*/*z* and ion intensities rather than cell-type marker genes. Assigning putative cellular origins to MSI signals therefore requires cross-modal label transfer and should be accompanied by uncertainty estimates and orthogonal validation [37].

Spatial-neighborhood association and label transfer: Registered cell boundaries and sampling footprints link cellular composition to ion signals. Finer ST measurements can be aggregated over MSI footprints, while finer MSI signals can be aggregated over ST units [54]. TACCO transfers compositional annotations by optimal transport between observations and a labeled reference in a common molecular feature space [85]. Its transcriptomic applications support cell annotation and mixture decomposition. Gene-to-ion transfer additionally requires a biological or experimentally learned cross-modal representation. Spatial overlap links cellular composition to ion signals at the measured scale, where intracellular, extracellular, and mixed-cell contributions may coexist.

Enzyme-informed metabolite attribution: Cell-type-specific expression of metabolic enzymes and transporters from scRNA-seq or SCP can be used to estimate probabilistic associations among cell types, enzymes, and ion features while retaining the confidence assigned to each candidate metabolite [37]. This is a hypothesis-generation step: Transcript or protein abundance alone does not determine reaction direction, flux, metabolite transport, or the cellular source of an MSI signal. Transcript-informed candidate ranking provides a biological prior for annotation, so chemical confirmation requires independent analytical evidence.

#### Mechanistic model construction and *in situ* validation

Following registration, deconvolution, and niche definition, spatial multi-omics analysis can move from description toward mechanistic hypothesis generation [86,87]. Integration of ST, imaging-based spatial proteomics, and MSI can support models that relate gene expression and protein abundance to detected ion features or validated metabolites across aligned tissue regions. Knowledge-guided integration uses prior biological relationships to support feature selection, prediction, clustering, and dimensionality reduction [88]. Frameworks such as Causal Oriented Search of Multi-Omics Space (COSMOS) incorporate prior biological knowledge to rank mechanistic hypotheses, but their outputs remain model-dependent and require experimental testing [89]. Accordingly, integrated analyses should distinguish statistical association, mechanistic plausibility, and experimentally demonstrated causality [86,87].

##### Cross-layer association and spatial graph construction

Joint embeddings and regulatory inference: SpatialGlue combines modality-specific graph embeddings through attention for joint domain discovery [57]. MultiGATE adds cross-feature priors and a 2-level graph attention autoencoder, whereas STARNet uses heterogeneous hypergraphs for RNA–chromatin regulatory inference [57,90,91]. SpatialGlue and MultiGATE require paired matrices at corresponding locations, so sampling scales must be matched beforehand and cross-sample batch effects addressed separately. Their neighborhood smoothing can strengthen shared structure while obscuring boundaries or modality-specific signals. MultiGATE has been evaluated for ST–metabolomics domain identification, whereas its strongest validation of regulatory relationships concerns genomic data. SpatialGlue has also been tested as a comparator on paired MSI–ST datasets, so evidence for recovering spatial domains should be distinguished from validation of individual gene–metabolite relationships [67]. Feature priors and attention-derived associations therefore require independent validation appropriate to the molecular layers being linked.

Local spatial association: Moran’s I and Geary’s C characterize within-layer spatial autocorrelation [92]. Spatially structured transcripts provide candidate features for downstream cross-modal association tests [92]. MANTIS, reported as a preprint, tests relationships in already registered ST–MSI profiles using spatial cross-correlation, partial correlation, and autocorrelation-preserving permutations, with optional regional or cell-type annotations for confounding control [93,94]. Evaluation should combine spatial-null calibration, multiple-testing control, and replication across specimens, including sensitivity to neighborhood definitions and registration error at tissue boundaries.

Heterogeneous graph representations: Spatial units and molecular features can form distinct node types linked by proximity or prior molecular relationships [91], enabling cross-layer module discovery [90,95]. Validation should match the intended output: held-out landmarks for alignment, independent markers or counts for composition, and held-out genes or specimens for reconstructed expression and joint representations. Spatial-block or whole-specimen holdouts reduce leakage between neighboring locations. Cross-sample evaluation should assess batch mixing alongside biological conservation, especially when batch and disease status overlap. Chemical assignments require analytical confirmation, and proposed mechanisms require functional testing.

##### Spatial metabolic-flux modeling of the gene–enzyme–metabolite axis

Gene expression can constrain hypotheses about enzyme abundance, but metabolic flux also depends on protein abundance, catalytic capacity, substrate availability, thermodynamics, and allosteric regulation. Evaluation of a gene–enzyme–metabolite axis therefore requires spatial molecular profiles to be integrated with genome-scale metabolic models (GEMs) and, where possible, direct measurements of metabolites or isotope incorporation.

Spatial flux balance analysis: Spatial transcriptomic or proteomic measurements can be used to derive context-specific constraints for a GEM. The Genome-scale metabolic model with Enzymatic Constraints using Kinetic and Omics data (GECKO) framework incorporates protein abundance and catalytic information into reaction bounds, although transcript abundance should not be treated as a direct substitute for enzyme capacity [96]. Flux balance analysis (FBA) then estimates feasible steady-state flux distributions under specified stoichiometric constraints and objective functions. In spatial flux balance analysis (spFBA), spatially resolved expression is used to adjust reaction bounds for individual spots, and sampling is used to obtain relative flux estimates across tissue regions [97]. These estimates are conditional on the model assumptions, and multiple flux distributions can satisfy the same steady-state constraints. Transcript- or protein-derived bounds constrain the solution space without independently validating its fluxes. Objective functions and constraint-mapping rules must be justified for the tissue and cell state under study; sensitivity analyses are needed because different assumptions can yield different flux patterns [97].

Comparison with MSI measurements: Predicted spatial flux patterns can be compared with MSI ion patterns assigned to candidate substrates and products, with key identities chemically validated [97,98]. For example, concordance among lactate dehydrogenase A (LDHA) expression, enzyme abundance, and chemically validated lactate- and pyruvate-associated ion-intensity patterns can be evaluated within registered regions [97,98]. Such comparisons can reveal discrepancies consistent with posttranslational modifications (PTMs), allosteric regulation, transport, or unmeasured reactions [99,100]. Conventional MSI maps relative ion abundance and does not directly measure flux, reaction rates, or turnover. Isotope-informed flux estimation combines a defined labeling design and sampling interval with model constraints, whereas targeted perturbation tests causal dependence [97,98]. Agreement with spatial abundance supports consistency with a proposed mechanism and can prioritize regulatory steps for testing; it does not establish a unique mechanism or directly validate flux. Isotope tracing, temporal measurements, independently measured kinetic parameters, and perturbation provide complementary evidence. Schwaiger-Haber *et al*. [98] combined MSI with stable-isotope labeling and spatial isotopologue spectral analysis to estimate fractional fatty-acid synthesis and elongation, with interpretation constrained by the labeling period and model assumptions.

##### Mechanistic modeling of spatial gradients and intercellular metabolic crosstalk

In the TME and in inflammatory lesions, metabolites can form spatial concentration gradients that contribute to intercellular signaling and metabolic exchange.

Reaction–diffusion modeling: Reaction–diffusion partial differential equations (PDEs), based on Fick’s second law, can model the transport of lactate, adenosine, or other diffusible metabolites from producing cells to neighboring consumers or responders [101,102]. These models represent cells or tissue regions as sources and sinks and can be fitted to spatial measurements, provided that secretion, uptake, degradation, and boundary conditions are specified explicitly [101]. In highly glycolytic tumors, lactate released by tumor cells can be taken up through monocarboxylate transporter 1 (MCT1) by regulatory T cells (Tregs), promoting nuclear factor of activated T cells 1 (NFAT1) signaling and programmed cell death protein 1 (PD-1) expression [103]. Extracellular adenosine is generated through sequential adenosine triphosphate (ATP) hydrolysis by ectonucleoside triphosphate diphosphohydrolase 1 (CD39) and ecto-5’-nucleotidase (CD73) and can suppress CD8-positive T cell function through adenosine A2A receptor (A2AR) signaling [104]. Parameterizing a reaction–diffusion model with cell-type-specific secretion and uptake estimates can generate testable maps of paracrine metabolite transport, but inferred flux fields should be validated experimentally.

Parameter identifiability concerns whether diffusion, production or secretion, uptake, and degradation can be estimated separately from the available observations. A static MSI distribution generally constrains their combined spatial effect, with multiple parameter combinations yielding similar patterns. Regional ion suppression, extraction, and matrix effects also complicate the relationship between MSI ion intensity and absolute tissue concentration. Conceptual models can explore candidate source–sink arrangements under explicit simplifying assumptions. Experimentally parameterized MSI-coupled models use independent transport or kinetic measurements, calibrated concentration data, or time-resolved observations to constrain the fitted parameters. Reports should distinguish externally measured, assumed, and fitted parameters and assess parameter uncertainty as well as sensitivity of model predictions. Competing source–sink or transport models should also be compared: A close fit to one static map may be shared by several mechanisms and therefore does not establish the fitted mechanism independently.

Parise *et al*. [105] paired a PDE-based hepatic sinusoidal model with MALDI-MSI of verapamil and its metabolites in rat liver. Parameterization used published multi-indicator dilution data and pharmacokinetic clearance information, while the MSI distributions were used to evaluate hepatic metabolic zonation.

Metabolite-receptor and transporter mapping: Spatial maps of normalized intensities for selected MSI *m*/*z* features, interpreted with their candidate identities and annotation confidence, can be compared with ST-derived expression of relevant transporters or receptors, such as MCT1, monocarboxylate transporter 4 (MCT4), and the adenosine A2A and A2B receptors (A2AR and A2BR) [106]. These maps represent relative detected-ion signals under the specified acquisition and preprocessing conditions. Absolute metabolite concentrations require calibrated measurements, while transport rates, receptor engagement, and metabolic flux require additional kinetic or functional evidence. Distance-weighted scores or optimal-transport models can then quantify the spatial association between a putative metabolite source and nearby responder-cell states [106,107]. Such scores may identify candidate metabolite-receptor–cell-state relationships, including associations with T cell dysfunction or macrophage polarization [106,108]. Targeted chemical analysis can resolve the candidate metabolites, while biochemical assays and perturbation test receptor engagement and causal effects.

##### *In situ* validation and perturbation testing

Computationally inferred networks should be evaluated with orthogonal imaging and functional perturbation experiments. Validation should test spatial colocalization, molecular specificity, and the predicted direction of effect.

Connecting MSI features to LC-MS/MS identities. Liquid chromatography–tandem mass spectrometry (LC-MS/MS) adds chromatographic separation and fragmentation evidence to an MSI candidate list. Matching proceeds by identifying candidates in the extract and then evaluating their connection to the MSI ion image. An evidence table links each MSI feature to the LC-MS/MS findings from matched tissue, recording alternative assignments and the spatial scale of validation.

1. Define the MSI ion and candidate set. Record feature ID, measured *m*/*z*, polarity, mass error, isotope evidence, proposed charge/adduct and molecular formula, and the regions in which the ion is detected. Check off-tissue and matrix blanks, and assess plausible isotopologues, fragments, and matrix adducts before treating the feature as an intact molecular ion [109]. Match candidates through the proposed neutral composition and ion chemistry: An MSI [M+Na]+ ion and an LC [M+H]+ ion can represent the same neutral molecule despite different *m*/*z* values. Mass tolerances should reflect calibration and mass resolving power in both datasets. Accurate-mass agreement narrows the candidate set, while structural identification draws on fragmentation and standard comparisons.
2. Analyze matched material by LC-MS/MS. Use an extraction and chromatographic method suited to the candidate class, for example, reversed-phase separation for many lipids or an appropriate polar-metabolite method. Document whether the extract comes from bulk tissue, a registered adjacent section, or a selected region, and include extraction blanks, quality controls, and suitable internal standards. Same-section extraction requires assessment of analyte recovery and compatibility with MSI preparation and sampling. Hendriks *et al*. [110] showed that prior MALDI-MSI affected subsequent protein identification and agreement between lipid quantification methods. Differences in extraction, ionization, and analytical coverage can explain why an MSI candidate is not detected by LC-MS/MS.
3. Resolve LC candidates before transferring annotations. For each compatible LC feature, examine precursor accurate mass, formula/adduct and isotope consistency, its extracted-ion chromatogram, and retention time. When several LC peaks share the mass and formula of an MSI feature, fragmentation and standard comparisons are used to discriminate among these chromatographic candidates. Conventional MSI has no chromatographic retention-time dimension: Retention time distinguishes compounds within the LC experiment and cannot be matched directly to an MSI pixel. Comparison with an authentic standard uses the same LC method to make retention times directly comparable. Literature or predicted retention times can help prioritize candidates [38,39].
4. Compare fragmentation and authentic standards. Acquire LC-MS/MS for the selected precursors and, where feasible, targeted on-tissue MS/MS in the relevant MSI region. Diagnostic product-ion masses and neutral losses are compared using compatible precursor/adduct, polarity, and fragmentation conditions because collision energy and co-isolation can affect spectral similarity. Class-specific lipid fragments support lipid-class annotation, while resolving individual isomers requires diagnostic structural evidence. For a key LC assignment, analyze an authentic standard under the same LC-MS/MS conditions and compare accurate mass, retention time, and MS/MS. Co-injection can further assess chromatographic agreement [38,39]. Library matches support putative compound annotations or lipid assignments at the structural level resolved by the spectra. Positional and stereochemical assignments require additional methods when isomers coelute or produce similar routine fragmentation patterns [47].
5. State the confidence of the MSI-to-LC connection. An authentic-standard-confirmed LC compound establishes its identity in the extracted material. Assigning that identity to an MSI ion image requires evidence that distinguishes it from other plausible contributors. Concordant on-tissue diagnostic fragments and region-specific LC results strengthen this connection. LC-MS/MS support is reported separately from MSI structural confirmation, and chemically plausible isomers remain in the candidate set for an MS1-based assignment. The evidence table should link MSI feature ID, measured ion/formula, LC feature ID and retention time, precursor and MS/MS records, standard evidence, specimen/region provenance, and separate LC-identity and MSI-assignment confidence. The table can link one MSI feature to several LC candidates or one compound to multiple supported ion forms.

MSI-guided LCM/LMD–LC-MS/MS validation. MSI ion images or molecular segmentation can first define spatially distinct regions of interest (ROIs), such as a molecular hotspot and a comparison region. Register the MSI map and optical or histological landmarks to a compatible laser-capture or laser-microdissection (LCM/LMD) section, verify alignment at the ROI scale, and record the cut boundaries and collected tissue amount [111]. For same-section sampling, assess analyte recovery; for adjacent sections, select corresponding ROIs and document section-to-section mismatch. Extract the low-input samples with controls for contamination and recovery, then perform LC-MS/MS using the candidate-matching and standard comparisons above.

Regional MSI-to-LC comparison. Compare LC abundance patterns with MSI summaries from corresponding ROIs after normalization for collected tissue amount and measurement-specific signal scaling. LCM preserves regional context by collecting selected tissue areas, so the LC-MS/MS result represents the sampled group of cells. Tissue amount, alignment accuracy, analyte recovery, and redistribution during preparation influence the sensitivity and spatial specificity of this comparison. Concordant patterns across ROIs strengthen the molecular assignment and regional localization. Resolving isomers at individual pixels requires spatially resolved chemical evidence; flux and causal mechanisms require tracing and perturbation.

Image-guided regional multi-omics: ROI sampling also provides a route to spatial multi-omics. MSI-defined zones or histological compartments can guide LCM/LMD for LC-MS/MS-based metabolomic, lipidomic, or proteomic profiling, using class-appropriate extraction and acquisition. Hendriks *et al*. [110], for example, combined MSI-guided dissection with lipidomic and proteomic LC-MS/MS analysis of the same tissue area. Linking these profiles to regional MSI and ST summaries through shared ROI identifiers and coordinates supports deeper molecular characterization and targeted structural validation within defined tissue regions. The sampled ROI is the cross-modal matching unit.

Multimodal high-resolution imaging: Single-molecule fluorescence *in situ* hybridization (smFISH) or RNAscope can be combined with multiplexed protein imaging, such as co-detection by indexing (CODEX; marketed as PhenoCycler) or imaging mass cytometry (IMC), and with high-resolution MSI to compare transcripts, proteins, and metabolites in registered tissue regions [112–116]. Same-section or serial-section workflows can visualize targeted RNA transcripts together with multiplexed protein markers, although the number of targets and the spatial resolution depend on platform and specimen preparation [112,113]. Fluorescence-based cell boundaries or fiducial signals can provide reference information for assigning MSI pixels to individual cells [114]. Ultrahigh-field (21-tesla) MALDI-FTICR MS can resolve chemical features at single-cell and subcellular scales [115]. The single-cell spatial metabolomics (scSpaMet) workflow combines ToF-SIMS with IMC to associate metabolite-related ion and fragment profiles with cell-type-specific protein markers [116]. Together, these methods relate transcripts, proteins, and ion signals within registered tissue regions. RNA and protein images provide cellular context, and complementary chemical measurements establish ion identity, with interpretation guided by registration accuracy and molecular coverage.

Perturbation-based validation: Candidate mechanisms can be tested in tissue models or patient-derived organoids using small-molecule inhibitors, neutralizing antibodies against programmed death-ligand 1 (PD-L1), or clustered regularly interspaced short palindromic repeats (CRISPR)-based gene editing; organoid and spatial causal-modeling studies illustrate complementary experimental and computational components of this strategy [117,118]. Models such as SpatialProp can predict tissue-level responses to multi-gene or multi-cell-type perturbations [119], whereas Celcomen generates counterfactual spatial-transcriptomic predictions [118]. These outputs are hypotheses to be tested, not substitutes for perturbation data. Patient-derived organoids have been used to investigate resistance to third-generation epidermal growth factor receptor tyrosine kinase inhibitors [117], and matched biopsy-organoid systems have linked microenvironmental cell states to drug response [120]. A causal conclusion requires reproducible intervention effects, appropriate controls, and validation across independent models; current evidence remains primarily methodological and proof-of-concept.

Method selection follows the intended output. Registration and footprint matching establish coordinate correspondence. Reference-based deconvolution estimates cellular composition, while joint representation learning can identify domains supported by multiple molecular layers. Testing a specific gene–ion relationship requires association analysis with spatial and biological controls. Table 1 summarizes these choices. Evaluation on paired data establishes performance only for the task and datasets tested, rather than validating every downstream chemical or mechanistic interpretation.

### Applications in tumor–host interface remodeling

The tumor–host interface is a transition zone in which malignant, immune, vascular, and stromal cells encounter gradients of oxygen, acidity, nutrients, extracellular matrix, and paracrine mediators. Spatial multi-omics has resolved regionally segregated adaptive cell states in glioblastoma and distinct stromal microenvironments in pancreatic ductal adenocarcinoma (PDAC). These measurements reveal tissue neighborhoods and their cellular composition, while same-section platforms align transcript and metabolite signals within a shared tissue plane [37,121,122].

Each modality contributes a different part of the evidence. MSI localizes ion features whose candidate identities are evaluated by chemical validation, whereas ST identifies cells or regions with the capacity to produce, transport, or respond to a metabolite; multiplexed protein imaging establishes cellular context; and tracer or kinetic measurements support flux estimates, while controlled perturbation tests functional dependence and causal direction. Claims about secretion, uptake, or reciprocal exchange require these dynamic and functional measurements in addition to spatial abundance maps. The examples below distinguish direct spatial observations from mechanistic evidence obtained in cell and animal models. The principal candidate mechanisms and the corresponding validation chain are summarized in Fig. 3.

**Fig. 3.**
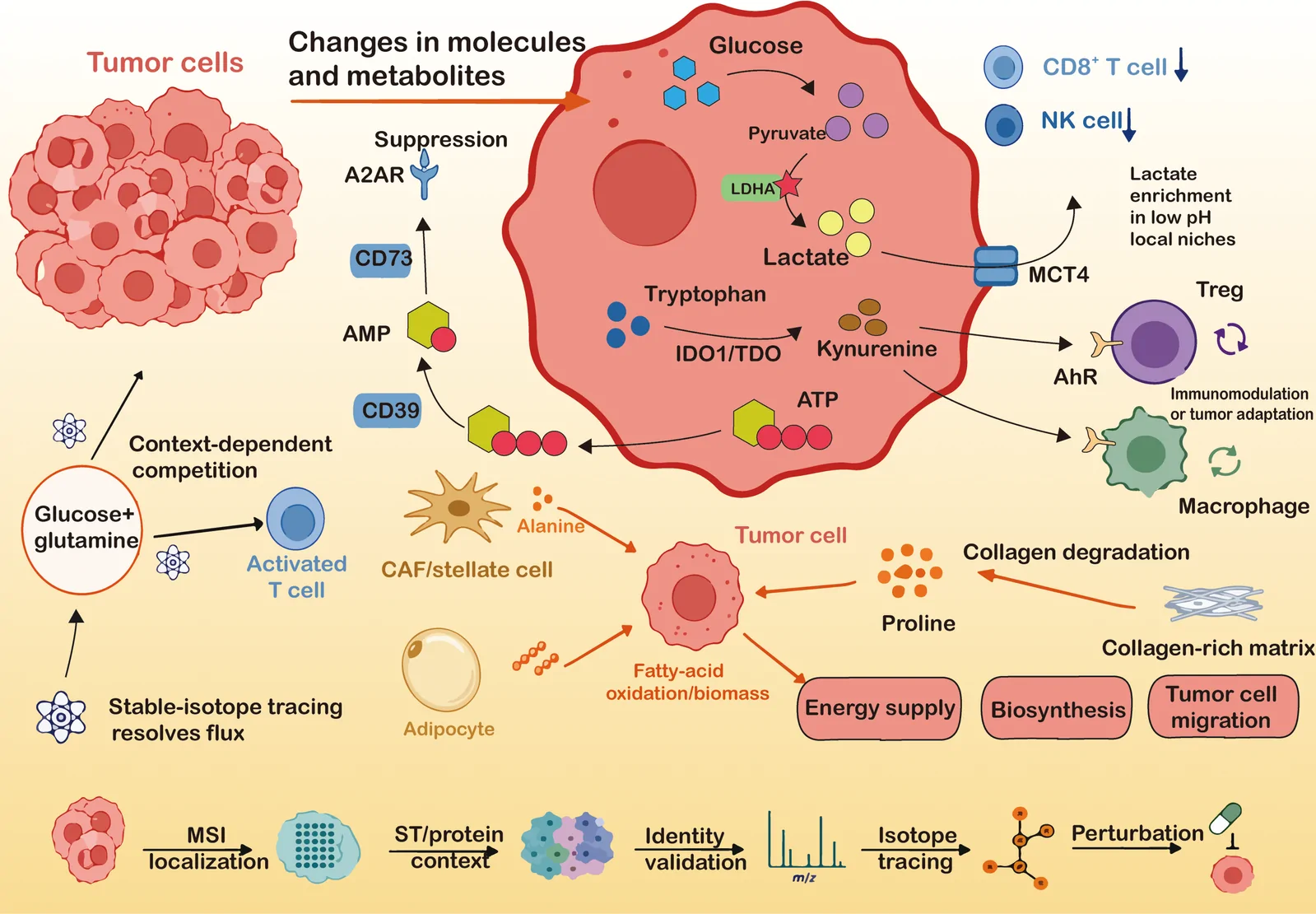
Metabolic interactions at the tumor–host interface and approaches to their validation. Tumor-cell glycolysis can generate lactate through LDHA and export it through MCT4, contributing to acidic niches that may impair CD8-positive T cells and natural killer cells and support regulatory T cell or macrophage adaptation. Parallel pathways include ATP conversion by CD39 and CD73 to adenosine with A2A-receptor signaling, and tryptophan catabolism by indoleamine 2,3-dioxygenase 1 or tryptophan 2,3-dioxygenase to kynurenine with aryl-hydrocarbon-receptor signaling. Nutrient partitioning, stellate-cell alanine release, adipocyte-derived fatty acids, and collagen-derived proline illustrate metabolic interactions between tumor cells and surrounding tissue. The lower workflow connects MSI localization and ST/protein context with chemical identity validation, isotope tracing, and perturbation. “Identity validation” involves comparing targeted MS/MS spectra with those of authentic metabolite or lipid standards under comparable precursor/adduct and fragmentation conditions. When LC-MS/MS is used, the comparison also includes retention time under the same chromatographic method. Isotope tracing provides information about metabolic flux and transfer, while perturbation tests the functional role of the proposed interactions. LC-MS/MS, liquid chromatography–tandem mass spectrometry; MSI, mass spectrometry imaging; MS/MS, tandem mass spectrometry; ST, spatial transcriptomics.

#### Diffusible metabolic and paracrine immunosuppressive niches

Lactate and extracellular acidification. Tumor-derived lactate can impair T and natural killer cell function and support immunoregulatory macrophage and regulatory T cell states [123–125]. Spatial measurements can identify where these populations coincide with chemically validated lactate distributions or acidic regions. Comparing MSI with ST or protein images can locate candidate neighborhoods, with appropriate normalization and independent lactate or pH measurements supporting gradient interpretation. Tracing and perturbation then test transport and functional dependence.

Adenosine and tryptophan catabolites. Adenosine signaling through the A2A adenosine receptor (A2AR) can suppress antitumor T cell function [126]. Kynurenine generated by tryptophan catabolism can activate the aryl hydrocarbon receptor and support tumor-associated immune suppression [127]. Spatial maps of CD39/CD73, receptors, and tryptophan-catabolizing enzymes identify regions with the molecular machinery for these pathways. Paired, chemically confirmed adenosine or kynurenine measurements can test whether these regions also show local metabolite enrichment. Adenosine stability requires careful sampling, while functional assays establish receptor engagement and causal effects.

A “metabolic shield” is a functional hypothesis linking a localized metabolic state to a responding cell population. Spatial measurements define the proposed barrier and its cellular context. Perturbing metabolite production, transport, or receptor signaling then tests whether that localized state contributes to immune suppression.

#### Nutrient partitioning and competitive metabolism

Nutrient availability at the interface reflects perfusion, diffusion, cellular uptake, extracellular-matrix transport, and local biosynthesis. Cell-type-resolved isotope studies have demonstrated programmed partitioning of major nutrients among malignant and immune compartments, while experimental work has shown that competition for glucose can restrict T cell effector function [128,129]. A low metabolite signal in an MSI image, however, cannot by itself identify the consuming cell type: The same pattern may result from ischemia, analyte loss during processing, ion suppression, rapid turnover, or differences in cell density.

Glutamine and amino-acid availability. Pharmacological glutamine blockade can impose divergent metabolic programs on cancer and immune cells in preclinical models [130]. Spatial integration can locate glutamine-associated ion patterns relative to cellular uptake programs. Chemical validation and independent concentration assays characterize local glutamine abundance; cell-type-resolved isotope tracing and transporter perturbation can identify the populations responsible for uptake.

Lipid acquisition and remodeling. Adipocyte-to-tumor lipid transfer at the omental ovarian-cancer interface has been linked to increased fatty-acid oxidation and tumor growth [131]. MSI linked to adipocyte and tumor-cell maps can locate lipid-associated signal enrichment at this interface. Lipid names should reflect the structural detail resolved. Labeled substrates and donor-cell perturbations then test transfer, while tracer or kinetic measurements resolve its rate and direction.

#### Stromal–metabolic reciprocity and translational interpretation

Spatial analysis can also locate candidate sites of stromal nutrient support. In PDAC models, pancreatic stellate cells supply alanine through autophagy-dependent secretion, and collagen-derived proline can support cancer-cell survival under nutrient limitation [132,133]. Spatial studies add the organization of stromal sub-tumor microenvironments and their associations with tumor-cell state and drug response [120,122,134]. Matched metabolite measurements and cell-type-resolved tracing can test whether local nutrient exchange contributes to these neighborhood-specific associations.

Therapeutic interpretation should likewise remain proportional to the evidence. Inhibition of lactate transport, glutamine metabolism, adenosine signaling, or stromal nutrient supply can be used to test a nominated interface mechanism in experimental systems. A spatially enriched pathway may justify biomarker-stratified preclinical testing, but it does not establish clinical efficacy or identify an optimal combination regimen. Drug response should be linked to target engagement in the same spatial compartment, assessed in independent models, and separated from nonspecific effects on proliferation or tissue viability.

MS-based intraoperative tissue-classification technologies provide a distinct translational route. DESI-MSI generates spatial ion maps and has classified gastric-cancer resection margins, whereas rapid evaporative ionization mass spectrometry (REIMS) and the handheld MasSpec Pen support rapid tissue classification through electrosurgical aerosol analysis and local probe sampling, respectively [135–137]. These studies demonstrate analytical and diagnostic feasibility; they do not yet show that a molecularly extended resection margin reduces recurrence or improves survival. Prospective trials should therefore compare the locked assay with standard pathology, report indeterminate measurements, and evaluate patient-relevant outcomes.

The tumor examples illustrate 4 complementary forms of evidence. Descriptive evidence establishes spatial distributions with reported molecular-identification confidence. Associative evidence links these patterns to cellular neighborhoods and reproducible covariance across specimens. Mechanistic plausibility arises from the location and molecular capacity of the relevant cells, while tracing and controlled perturbation test transport, flux, and causal dependence. Translational evidence adds analytical validity, performance for a prespecified clinical use, and demonstrated clinical utility. These forms of evidence and the principal spatial programs operating at tumor–host interfaces are summarized in Table 2. Their relevance depends on the proposed claim rather than a fixed sequence of validation steps.

**Table 2.** Evidence-constrained framework for metabolic remodeling at tumor–host interfaces. Direct spatial evidence is distinguished from mechanistic evidence obtained in cell, organoid, or animal models.

Tumor or interface context	Candidate spatial program	Direct spatial evidence and platform	Mechanistic evidence	Interpretation and translational relevance	Evidence boundary and required validation
Glioblastoma (GBM)	Regionally segregated reactive, inflammatory, and metabolic tumor states coupled to host-cell composition.	Spatial transcriptomics integrated with metabolomic imaging and imaging mass cytometry identified locoregional tumor–host interdependence and spatially exclusive adaptive programs.	Human and rodent neocortical models supported environmental induction of tumor-cell state rather than a purely fixed genetic explanation.	Supports niche-aware stratification and nomination of region-specific dependencies; demonstrates a feasible multimodal validation architecture.	Not every detected ion was structurally confirmed, and the study does not establish a specific diffusible lipid or lactate halo. Replication in independent cohorts, on-tissue tandem mass spectrometry, and pathway-specific perturbation are needed.
PDAC stromal sub-tumor microenvironments	Spatially confined “reactive” and “deserted” stromal states; tumor epithelial state varies with local fibroblast and immune composition.	Histology-guided spatial profiling, single-cell analyses, and tissue architecture studies demonstrate reproducible stromal organization.	Matched biopsy–organoid experiments show that microenvironmental context can alter tumor-cell state and drug response.	Supports spatially stratified sampling and testing of microenvironment-dependent vulnerabilities.	These data establish spatial organization and plasticity, not a directly measured metabolite gradient. Same-region metabolomics, cell-resolved protein validation, and functional intervention are required before assigning a metabolic exchange mechanism.
Lactate-rich/acidic niches across solid-tumor models	High glycolytic activity, lactate export, extracellular acidification, and altered immune-cell function.	Direct spatial lactate measurements are study- and platform-dependent; lactate dehydrogenase and transporter expression are only pathway-capacity markers.	Tumor-derived lactate suppresses T and natural killer cells, promotes immunoregulatory macrophage states, and supports regulatory T cells in experimental systems.	Justifies testing lactate production, transport, or pH regulation as compartment-specific mechanisms; may guide pharmacodynamic sampling.	A universal radial “lactate shield” is not established. Confirm lactate by authentic standard/tandem mass spectrometry, measure pH and tissue concentration independently, and demonstrate phenotype reversal after production or transport blockade.
Adenosinergic immune-suppression niche	Stress-associated nucleotide release; CD39–CD73 conversion to adenosine; A2A adenosine receptor signaling in immune cells.	Spatial mapping generally measures ectonucleotidases, receptors, or transcript signatures more robustly than extracellular adenosine itself.	Genetic deletion or blockade of A2A adenosine receptor signaling can release antitumor T cell activity in tumor models.	Supports biomarker-guided experimental evaluation of CD39, CD73, or adenosine-receptor interventions.	Enzyme/receptor coexpression is not a measured adenosine halo. Rapid specimen handling, direct chemical validation, extracellular-context assays, and receptor-specific perturbation are required.
Tryptophan–kynurenine niche	Indoleamine 2,3-dioxygenase 1 or tryptophan 2,3-dioxygenase activity; kynurenine production; aryl hydrocarbon receptor activation.	Spatial enzyme expression can nominate candidate regions, but an accurate-mass feature alone cannot uniquely identify kynurenine or establish extracellular depletion.	Tumor-derived kynurenine can activate the aryl hydrocarbon receptor and promote tumor-supportive programs.	Supports paired pathway measurements and mechanistic testing; may inform selection of downstream rather than enzyme-only interventions.	Measure tryptophan and kynurenine together, confirm chemical identity, and assess aryl hydrocarbon receptor activity. Enzyme redundancy and negative clinical experience with some indoleamine 2,3-dioxygenase 1 strategies preclude simple extrapolation.
Glutamine-partitioning niche	Cell-type-specific glutamine uptake and use; competition or differential adaptation among malignant and immune cells.	Isotope-based cell-type-resolved studies support nutrient partitioning; MSI intensity alone does not identify extracellular concentration or consuming cell type.	Glutamine blockade can impose divergent programs on cancer and immune cells in preclinical models.	Supports testing context-specific glutamine dependencies and immune effects rather than assuming uniform nutrient deprivation.	Distinguish reduced delivery from increased uptake. Use stable-isotope tracing, transporter/enzyme perturbation, perfusion measures, and independent concentration assays; report tumor model and treatment context.
PDAC fibroblast–tumor cross-feeding	Stellate-cell alanine secretion and extracellular-matrix-derived proline as alternative carbon/nitrogen sources under nutrient limitation.	The original evidence combines conditioned medium, isotope tracing, cell culture, and animal models rather than direct human spatial metabolite imaging.	Autophagy-dependent alanine release and collagen-derived proline utilization support cancer-cell metabolism in defined PDAC models.	Provides tractable donor–recipient hypotheses for spatial tracing and stromal-targeted intervention.	Human relevance and compartment specificity require confirmation. Demonstrate donor origin, recipient uptake, pathway dependence, and rescue; avoid inferring transfer from adjacent expression alone.
Ovarian cancer–omental adipocyte interface	Adipocyte lipolysis, fatty-acid transfer, tumor-cell fatty-acid oxidation, and interface-associated fatty-acid-binding protein 4 expression.	Histology localized lipid-rich cancer cells at the adipocyte interface; coculture and *in vivo* experiments demonstrated transfer and functional dependence.	Adipocyte-derived lipids promoted ovarian-cancer growth, and fatty-acid-binding protein 4 deficiency impaired metastatic growth.	Supports a context-specific donor–recipient lipid network and biomarker-guided investigation of lipid transport.	This is a peritoneal metastatic niche, not a universal invasive-margin mechanism. Validate lipid species, transfer direction, and patient heterogeneity before generalizing to other tumors.
Molecular assessment of surgical margins	Lipid/metabolite spectral patterns used to distinguish tumor from nontumor tissue during or near the time of surgery.	Desorption electrospray ionization MSI, rapid evaporative ionization mass spectrometry, and handheld sampling have shown tissue-classification feasibility.	Diagnostic models associate multivariate spectra with reference histopathology; these are classification studies rather than interface-flux studies.	Potential adjunct for rapid margin triage, targeted sampling, or intraoperative decision support.	Requires locked algorithms, external/multicenter validation, prospective comparison with pathology, accounting for indeterminate spectra, and clinical-utility outcomes. Diagnostic accuracy does not itself prove reduced recurrence.

A2AR, adenosine A2A receptor; AhR, aryl hydrocarbon receptor; GBM, glioblastoma; MSI, mass spectrometry imaging; PDAC, pancreatic ductal adenocarcinoma; ST, spatial transcriptomics

### Applications and emerging opportunities beyond oncology

Beyond oncology, spatial multi-omics is particularly useful in organs whose normal function is organized along anatomical gradients. Oxygen tension, nutrient delivery, vascular supply, and local signaling partition tissues into functional compartments; pathology may therefore emerge through selective remodeling of these compartments rather than through uniform molecular change. Metabolic dysfunction-associated steatotic liver disease (MASLD) is the best-developed example, with recent human and experimental studies combining ST and spatial metabolomics to resolve zonal lipid remodeling and fibrosis-associated cellular states [138,139]. In adipose tissue and reproductive disease, spatial transcriptomic and imaging studies have mapped progenitor, immune, and stromal niches, while paired metabolite-resolved datasets remain scarce. These settings therefore offer emerging opportunities for MSI integration, with existing cellular and anatomical maps defining where metabolite measurements could be most informative.

#### MASLD: Remodeling of hepatic zonation and fibrosis-associated niches

The hepatic lobule supports a continuous porto-central division of labor. Periportal regions favor oxidative metabolism, gluconeogenesis, and ureagenesis, whereas pericentral regions are enriched for lipogenesis and xenobiotic metabolism. These patterns arise from combined oxygen and nutrient gradients and from zonated signaling; they also provide a spatial reference against which disease-associated change can be interpreted [139].

Human data now show that MASLD does not remodel this architecture uniformly. Li *et al*. [138] profiled 61 livers spanning control, steatosis without steatohepatitis, and metabolic dysfunction-associated steatohepatitis (MASH), using scRNA-seq, ST, and MALDI-MSI in a subset. The study identified stage-associated cellular programs and phospholipid-rich regions that were spatially coupled to pathological remodeling. Figure 4 summarizes the anatomical scaffold, disease-associated remodeling, and an analysis path from aligned ST–MSI measurements to testable mechanisms.

**Fig. 4.**
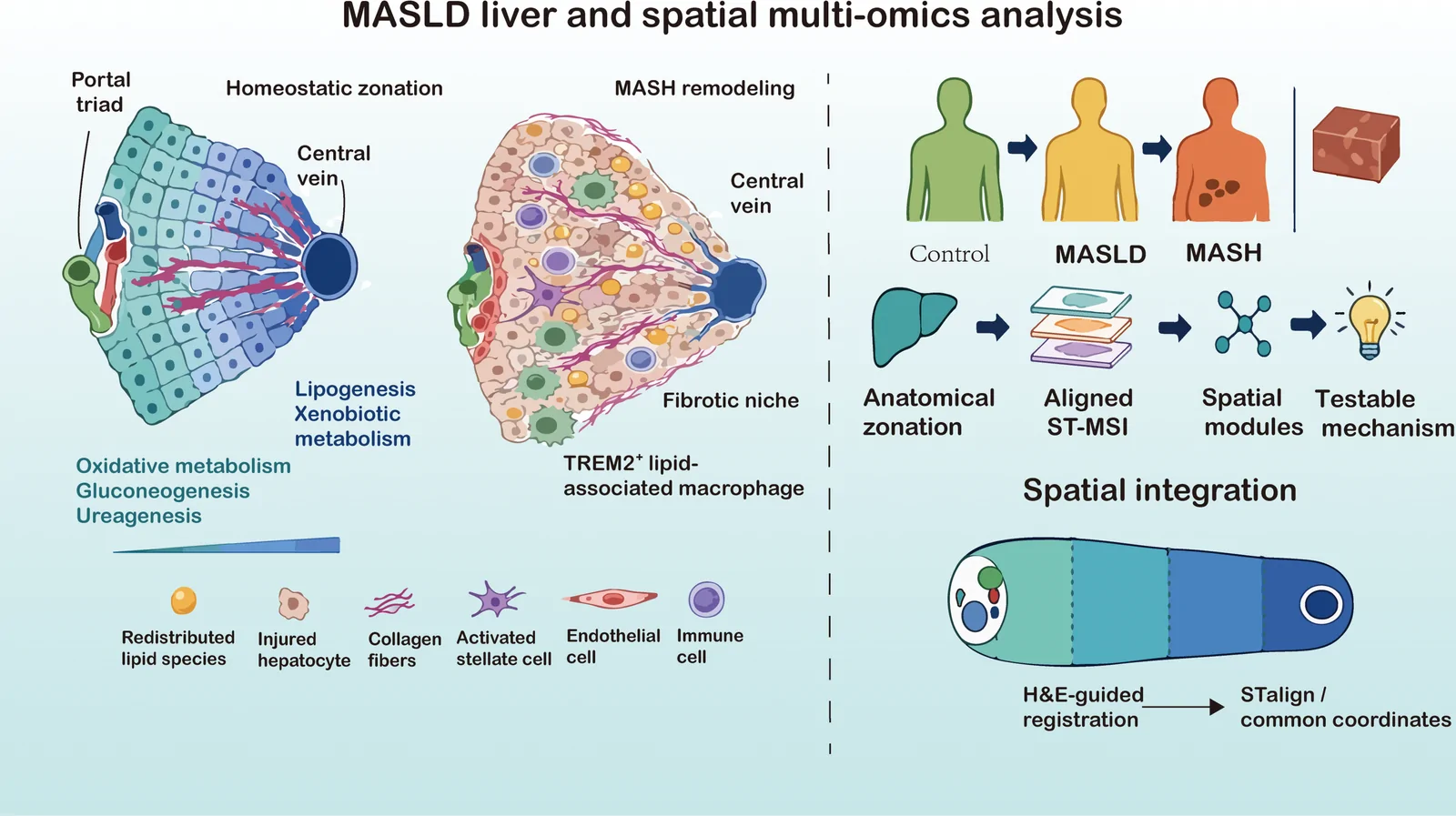
Hepatic zonation and a spatial multi-omics strategy for MASLD. The normal porto-central axis separates periportal oxidative metabolism, gluconeogenesis, and ureagenesis from pericentral lipogenesis and xenobiotic metabolism. During progression to metabolic dysfunction-associated steatohepatitis, lipid species are redistributed and injured hepatocytes, activated stellate cells, endothelial cells, immune cells, collagen-rich fibrotic niches, and TREM2-positive lipid-associated macrophages become spatially reorganized. Integration should preserve anatomical zonation, use H&E-guided registration of ST and MSI data, identify spatial modules, and nominate mechanisms for chemical and perturbational testing. Serial sections require explicit registration-error assessment, and lipid assignments should reflect the structural detail established by chemical validation. H&E, hematoxylin and eosin; MASLD, metabolic dysfunction-associated steatotic liver disease; MASH, metabolic dysfunction-associated steatohepatitis; MSI, mass spectrometry imaging; ST, spatial transcriptomics; TREM2, triggering receptor expressed on myeloid cells 2.

##### Zonal lipid remodeling

In a murine MASH model, Seubnooch *et al*. [140] reported 130 lipid species whose abundance differed by lobular zone. Several triacylglycerol, diacylglycerol, sphingolipid, and ceramide signals shifted from predominantly pericentral distributions toward periportal regions as disease progressed, indicating that steatotic remodeling includes redistribution as well as net accumulation. In this study, accurate-mass database assignments were refined by DESI-MS/MS and direct-infusion MS/MS, providing lipid annotations at the structural level resolved by the diagnostic fragments.

Otkur *et al*. [139] combined ST and spatial metabolomics on adjacent liver sections from high-fat-diet and G protein-coupled receptor 35 (GPR35)-deficient mouse models. Zone-dependent phospholipid patterns covaried with transcriptional programs related to lipid metabolism, and genetic perturbation altered these relationships. Because the work was performed in mice and used adjacent sections, it supports a zonated GPR35-associated phenotype but does not by itself establish the corresponding mechanism in human MASLD.

Interpreting individual lipid ions requires both chemical specificity and reliable signal measurement. Accurate *m*/*z* values may remain compatible with multiple isobaric or isomeric candidates [39]. Extraction and ionization efficiency also affect signal intensity. Lipid assignments central to a mechanistic claim should be supported by MS/MS, authentic standards where feasible, and orthogonal LC-MS/MS analysis [38]. The reported lipid name should specify the class, sum-composition, molecular-species, or more detailed structural level resolved by the analytical evidence [47]. Region-matched lipid validation can use MSI-guided microdissection followed by lipidomic LC-MS/MS [110]. Tracer or kinetic measurements are needed to distinguish production, uptake, transport, or impaired turnover [97,98].

##### Fibrosis-associated cellular niches

Fibrosis is likewise organized at the scale of local multicellular neighborhoods. Injured hepatocytes, hepatic stellate cells, endothelial cells, and recruited or resident immune cells occupy partially overlapping regions in which inflammatory and extracellular-matrix programs are coordinated. In the human atlas reported by Li *et al*. [138], fibrotic progression was accompanied by spatially restricted changes in parenchymal, stromal, and immune states rather than a single liver-wide response.

These maps refine, but do not replace, established models of fibrogenesis. They can show where stellate-cell activation, endothelial remodeling, and hepatocyte stress coincide; causal direction still requires perturbation in a model that preserves the relevant architecture. Serial-section integration also introduces registration uncertainty, so apparent cell–metabolite associations should be interpreted at the spatial scale supported by the assay.

One reproducible feature is the expansion of triggering receptor expressed on myeloid cells 2 (TREM2)-positive lipid-associated macrophage states in advanced disease. Li *et al*. [138] linked these macrophages to phospholipid remodeling and identified a candidate regulatory axis connecting microphthalmia-associated transcription factor (MITF) with hepatocyte growth factor (HGF). MITF gain- and loss-of-function experiments supported its role in macrophage programs, and conditioned-medium experiments supported a hepatoprotective effect associated with HGF secretion. These experiments support macrophage–hepatocyte signaling, while the turnover of individual imaged phospholipids and the clinical effects of targeting this axis remain unresolved.

Together, these studies provide direct spatial evidence for zonal lipid remodeling and associations with fibrosis-related cellular states. The GPR35 mouse experiments add perturbational support for a zonated phenotype, while the human atlas includes functional follow-up in cell models. Identifying a lipid mediator of that phenotype requires chemical confirmation and evidence of functional dependence, supplemented by tracer or kinetic measurements for proposed flux or transport mechanisms. Clinical utility remains a separate question for prospective human studies.

#### Adipose tissue: Spatial niches for future MSI integration

White adipose tissue (WAT) contains mature adipocytes, vascular and immune cells, and heterogeneous stromal progenitors distributed across lobules, septa, and vascular interfaces. Dissociation-based atlases resolve cell states but remove these relationships; ST and imaging can restore anatomical context. In human WAT, integration of single-cell, single-nucleus, and spatial transcriptomic data resolved more than 60 cell populations and placed distinct fibro-adipogenic progenitor states near endothelium or lipid-associated macrophages [141].

Progenitor hierarchy was first delineated in mouse adipose tissue by Merrick *et al*. [142], who used single-cell transcriptomics and histological validation to identify dipeptidyl peptidase 4 (DPP4)-positive multipotent progenitors in the reticular interstitium and more committed intercellular adhesion molecule 1 (ICAM1)-positive and tissue factor (CD142)-positive preadipocyte states. These transcriptomic and histological measurements establish anatomical localization and lineage organization; niche-specific lipid composition remains to be characterized by MSI.

Obesity also reorganizes immune neighborhoods. Stansbury *et al*. [143] combined scRNA-seq, ST, and imaging in diet-induced obesity and found precursor lipid-associated macrophage (pre-LAM) and lipid-associated macrophage (LAM) states associated with developing crown-like structures. Together, these studies define the spatial and cellular organization of progenitor and macrophage niches. Chemically validated, region-matched MSI measurements would connect those niches to specific metabolites, identifying candidates for subsequent tracer and perturbation studies.

#### Preeclampsia: Spatial frameworks for future MSI integration

Preeclampsia (PE) is a pregnancy-specific hypertensive syndrome associated with placental dysfunction and a systemic maternal response. Abnormal trophoblast invasion and spiral-artery remodeling are important components, but PE is heterogeneous and cannot be reduced to a single decidual defect. Spatial studies are therefore most informative when the sampled tissue, gestational stage, clinical history, and assay modality are stated explicitly.

Evidence for a maternal decidual contribution predates current spatial atlases. Garrido-Gomez *et al*. [144] found impaired decidualization in endometrial stromal cells obtained after pregnancy from women with a history of severe PE, suggesting a persistent maternal susceptibility. Because the samples were collected outside the affected pregnancy, the study supports an association with prior PE but does not map the active maternal–fetal interface.

Muñoz-Blat *et al*. [145] subsequently combined scRNA-seq with ST and laser-capture microdissection–proteomics in separate discovery and replication cohorts of late-secretory endometrium from women with and without a history of severe PE. They described mosaic decidualization resistance involving stromal and epithelial compartments, with altered steroid-response, WNT, and secreted phosphoprotein 1 (SPP1)-related programs. A separate atlas of healthy first- and second-trimester implantation sites provides a reference for trophoblast, stromal, vascular, and immune organization during normal placentation [146]. These studies establish the cellular and anatomical context of decidualization-related associations. Paired, chemically validated MSI could identify metabolites within these compartments for subsequent tests of metabolic mechanisms or biomarker utility in the relevant pregnancy setting. Figure 5 places these reproductive findings alongside the analytical workflow used to resolve adipose-tissue niches.

**Fig. 5.**
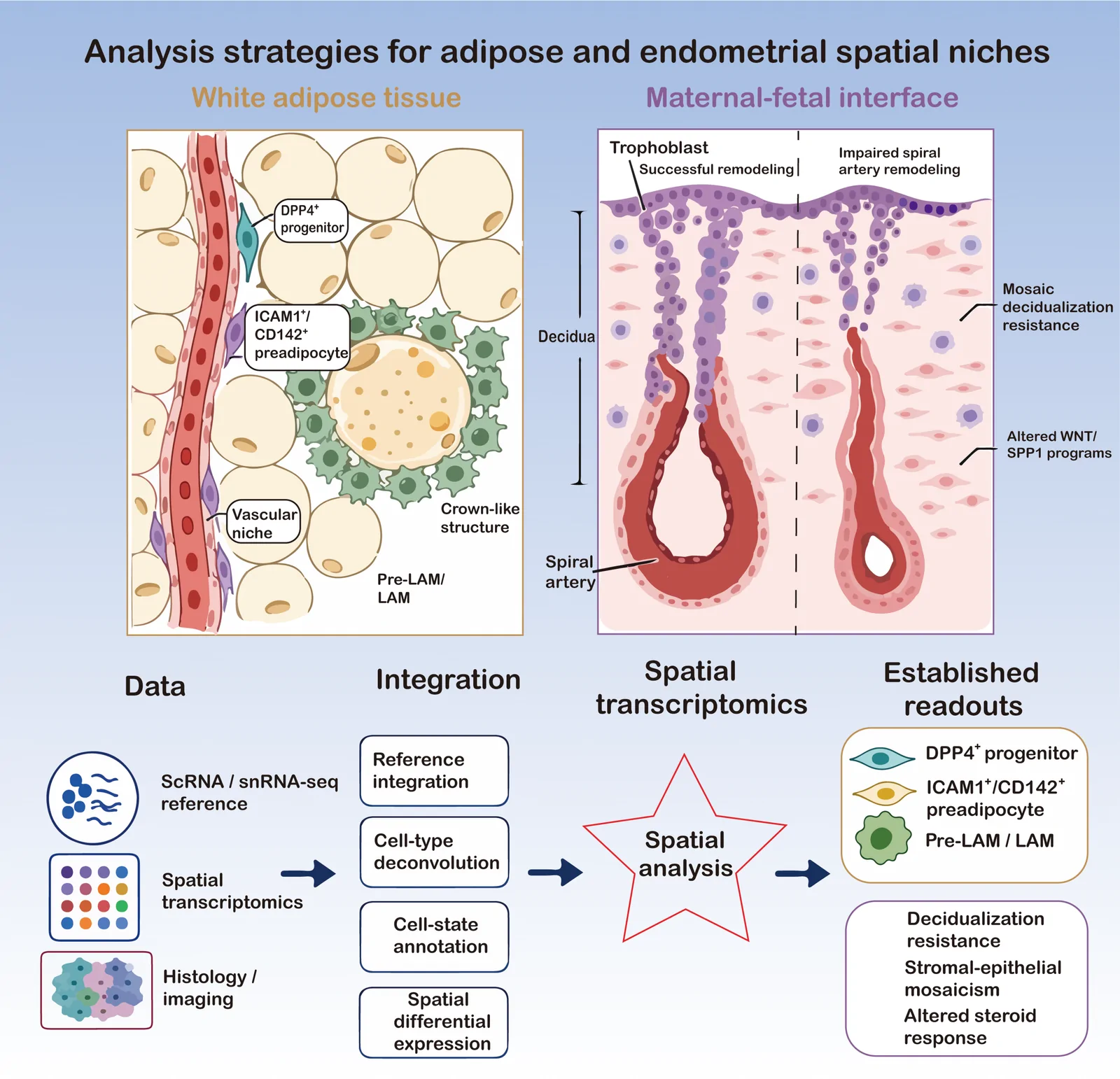
Conceptual overview of spatial niches in white adipose tissue and reproductive tissues. The adipose panel summarizes stromal progenitor states, vascular niches, and macrophage-rich crown-like structures. DPP4-positive progenitors include the reticular-interstitial population described by Merrick *et al*.; the illustrated cell positions are schematic. The reproductive panel places endometrial decidualization-related findings in the broader context of trophoblast invasion and spiral-artery remodeling. The lower panel presents an example analytical workflow combining single-cell or single-nucleus RNA-sequencing references, spatial transcriptomics, and histology or imaging. Reference integration, cell-type deconvolution, cell-state annotation, and spatial differential-expression analysis represent general analytical tasks. Their selection and implementation depend on the dataset and spatial platform. The illustrated readouts describe cellular organization and transcriptional programs, providing a framework for future metabolite-resolved MSI studies with orthogonal chemical validation. Mosaic decidualization resistance and altered WNT- and SPP1-related programs were identified in nonpregnant endometrium after severe preeclampsia, separately from observations at healthy implantation sites. Neither establishes a paired MSI–ST metabolic mechanism in active preeclampsia. CD142, tissue factor; DPP4, dipeptidyl peptidase 4; ICAM1, intercellular adhesion molecule 1; LAM, lipid-associated macrophage; MSI, mass spectrometry imaging; pre-LAM, precursor lipid-associated macrophage; scRNA-seq, single-cell RNA sequencing; snRNA-seq, single-nucleus RNA sequencing; SPP1, secreted phosphoprotein 1.

Across the applications, evidence should be matched to the claim under discussion. Table 3 separates molecular identity, spatial correspondence, cellular attribution, mechanistic plausibility, flux or transport, causality, and clinical utility. A study may provide strong evidence for one of these questions while leaving others open; absence of a flux measurement, for example, does not invalidate a well-supported localization result.

**Table 3.** Evidence status of representative applications. The 7 checkpoints are reported for the specific claims and experiments summarized here, not combined into a numerical score or a linear maturity ranking. “Not established” concerns the stated claim in the cited example, not the validity of all findings in that study. Regional associations do not assign an ion uniquely to a cell. Separate-cohort replication tests recurrence of a biological pattern rather than local molecular correspondence. Interpretation of paired data remains limited by the registration uncertainty reported for each dataset.

Application and study	Molecular identity and spatial correspondence	Cellular attribution and mechanistic plausibility	Flux or transport, causality, and clinical utility
Glioblastoma niches Ravi *et al*. [121]	Identity: ion features with candidate annotations. Spatial: MSI and ST on different sections from the same block; regional alignment remains limited by deformation and section heterogeneity.	Attribution: regional mixtures linked to cell states. Mechanism: transcript/protein context and experimental models support environmentally induced state changes.	Flux: specific metabolite exchange not established by the maps. Causality: state plasticity tested in models, not every ion–cell link. Utility: no demonstrated clinical benefit.
Human MASLD Li *et al*. [138]	Identity: annotated phospholipid signals with unresolved isomeric alternatives. Spatial: adjacent-section MSI–ST with landmark-guided alignment; correspondence requires sufficient histological similarity.	Attribution: regional lipid–cell-state associations; cellular markers provide supporting localization. Mechanism: MITF programs and HGF secretion have functional support in cell models.	Flux: cell respiration assays do not measure spatial phospholipid turnover. Causality: MITF perturbation and conditioned-medium assays support macrophage and hepatocyte functions; lipid-specific mediation remains unresolved. Utility: not established.
Mouse MASLD Otkur *et al*. [139]	Identity: formula/lipid candidates, including unresolved alternatives. Spatial: adjacent liver sections; zonal correspondence, not identical-cell matching.	Attribution: regional mixtures along the lobule. Mechanism: lipid patterns covary with enzyme and transcriptional programs under diet and Gpr35 perturbation.	Flux: zonal synthesis or transport rates not directly measured. Causality: gene deletion supports a GPR35-dependent phenotype; specific lipid mediation remains unresolved. Utility: no validated human application.
Mouse MASH lipid zonation Seubnooch *et al*. [140]	Identity: accurate-mass assignments refined by tandem MS, at the structural detail resolved. Spatial: zonal MSI linked to histology; not a paired MSI–ST study.	Attribution: lobular regions rather than individual producing or consuming cells. Mechanism: disease-associated lipid redistribution nominates pathways for testing.	Flux: not inferred directly from abundance. Causality: redistribution alone does not establish the responsible pathway. Utility: not established.
Adipose niches Massier *et al*. [141], Merrick *et al*. [142], and Stansbury *et al*. [143]	Identity: niche-specific metabolites not resolved by these datasets. Spatial: transcriptomic and imaging localization; no paired MSI–ST correspondence to evaluate.	Attribution: progenitor and macrophage states mapped to tissue niches. Mechanism: lineage and niche organization have cellular support; specific lipid exchange remains a hypothesis.	Flux: niche metabolite transfer unmeasured. Causality: cellular findings do not establish a metabolite-mediated mechanism. Utility: MSI-based clinical benefit not assessed.
Decidualization and prior severe PE Muñoz-Blat *et al*. [145]	Identity: transcript/protein evidence, not metabolite identification. Spatial: compartment profiling across separate discovery and replication cohorts; no paired MSI–ST map.	Attribution: stromal and epithelial states and regional programs. Mechanism: steroid-response and communication pathways supported by cross-cohort molecular findings.	Flux: not measured. Causality: associations in nonpregnant endometrium do not establish a metabolic cause of active PE. Utility: replicated features do not establish predictive or treatment benefit.
Molecular surgical-margin assessment Eberlin *et al*. [135]	Identity: discriminatory ion patterns; full structural identification is not required for every classifier feature. Spatial: DESI-MSI compared with reference histopathology.	Attribution: tissue classes or regions. Mechanism: a diagnostic classifier need not resolve the complete mechanism of its spectral differences.	Flux and causality: not required to evaluate tissue classification. Utility: classification feasibility does not demonstrate improved surgical decisions, recurrence, or survival; prospective outcome evaluation is needed.

MSI, mass spectrometry imaging; ST, spatial transcriptomics; MASLD, metabolic dysfunction-associated steatotic liver disease; MASH, metabolic dysfunction-associated steatohepatitis; PE, preeclampsia; MITF, microphthalmia-associated transcription factor; HGF, hepatocyte growth factor

## Technical Challenges and Future Directions

Combining MSI or single-cell mass spectrometry (SC-MS) with single-cell and ST links molecular composition to cell state and tissue architecture. The analytical layers, however, differ in sampling unit, dynamic range, missingness, spatial resolution, and molecular-identification certainty. These differences make integration a measurement problem as much as a computational one. Progress toward mechanism or clinical use therefore depends on transparent uncertainty, fit-for-purpose validation, and reproducible pre-analytical workflows.

### Data integration: Modality mismatch and molecular ambiguity

Transcriptomic data are discrete counts indexed by cells or capture spots, whereas MSI data are continuous ion intensities indexed by pixels and are influenced by matrix application, ion suppression, extraction, and instrument drift. The modalities may also be collected from the same section, adjacent sections, or different specimens. An integration model cannot recover cell-level correspondence that was not measured; its spatial claims must match the co-registration accuracy and effective resolution of the underlying assays [147].

Methods now include probabilistic latent-variable models, graph-based representations, and optimal-transport alignment. SpatialMETA, for example, was designed to integrate spatial transcriptomic and metabolomic data across samples and modalities, while topography-aware optimal alignment of spatially resolved tissues (TOAST) addresses spatial alignment without requiring identical features [67,148]. Such methods should be evaluated against known anatomical landmarks, held-out features, and sensitivity to registration error. A visually coherent latent space is not, by itself, evidence that inferred cross-modal relationships are biologically correct.

Molecular annotation determines the chemical specificity of MSI interpretation, independently of registration or integration accuracy. Many untargeted tissue MSI workflows primarily acquire MS1 spectra without chromatographic separation. Accurate precursor *m*/*z* and isotope patterns can constrain molecular formulas but often cannot uniquely distinguish isobaric candidates and cannot resolve structural or stereochemical isomers sharing a formula. Multiple adducts, isotopologues, in-source fragments, and, in MALDI, matrix-derived ions further complicate the relationship between peaks and endogenous molecules. Thus, one molecule can generate several ion features, while one unresolved feature can contain contributions from multiple species. FDR-controlled approaches such as METASPACE improve formula-level annotation, while discrimination among structural isomers requires additional chemical evidence [46].

Structural specificity must also match the evidence available for lipids. A sum-composition annotation such as phosphatidylcholine (PC) 34:1 does not determine its constituent fatty-acyl chains, their sn-positions, or double-bond positions and geometry. Routine MS/MS may support a lipid class or acyl-chain composition yet leave positional and stereochemical alternatives unresolved. Higher mass resolving power can separate some near-isobaric ions but cannot distinguish species with identical exact masses on that basis alone. Chromatography, ion mobility, and specialized fragmentation or derivatization can distinguish candidates through differences in retention, mobility, or diagnostic reaction and fragmentation products [47].

Structure-selective MSI strategies: Ion-mobility-enabled MSI adds a gas-phase separation dimension, allowing selected isobaric or isomeric ions to be mapped separately. Collision cross sections, matched adduct/charge states, MS/MS, and reference measurements support assignment of the separated features. Helmer *et al*. [149] combined MALDI-MSI with trapped ion mobility spectrometry (TIMS) and a chromatography-derived mobility database to improve phospholipid assignments. Diagnostic fragmentation such as ozone-induced dissociation, or targeted Paternò–Büchi derivatization followed by MS/MS, provides carbon–carbon double-bond localization for selected lipids. Method selection should match the structural question, with the reported lipid name reflecting the detail experimentally resolved [47]. This structural detail should be retained in confidence records and downstream pathway analysis.

For assignments central to biological interpretation, reporting should include measured *m*/*z*, polarity, mass error, proposed adduct and formula, database and version, candidate alternatives, and a stated confidence framework. On-tissue MS/MS and comparison with authentic standards should be complemented, where necessary, by LC-MS/MS or other orthogonal measurements that discriminate the proposed alternatives. Chemical validation identifies compounds in the sampled extract; assigning an unresolved isomer to a particular MSI pixel requires spatially resolved chemical evidence. Reporting therefore distinguishes extract-level identification, pixel-level assignment, and evidence of flux or causality [38,39].

Annotation uncertainty propagates into pathway assignment, metabolic interpretation, and cross-omics conclusions: Alternative candidates for the same ion may map to different reactions, and redundant adduct or fragment signals may inflate apparent pathway support if counted as independent metabolites. Enzyme expression or pathway context provides a biological prior for ranking candidates. Using that prior both to assign an ion and to validate the same transcriptomic pathway introduces circular inference. Analyses should retain plausible candidate sets, group related ion forms where justified, and test whether pathway or network conclusions survive alternative assignments. S2IsoMEr addresses molecular ambiguity through repeated sampling of candidate identities during enrichment analysis, while SpatialMETA recommends orthogonal LC–MS validation of key assignments [67,150].

A practical sensitivity analysis can follow one proposed association from the measured ion to its biological interpretation. For example, an MSI feature annotated as a phosphatidylcholine candidate may covary with a macrophage-rich ST region. Plausible candidate identities and ion-form groupings, mass tolerances and peak-alignment settings supported by calibration, and normalization choices supported by quality controls should be prespecified. These alternatives should be combined with registration perturbations calibrated to landmark errors, footprint aggregation at justified scales, and alternative cell-state or neighborhood definitions. The association should be refitted with the same patient-level partitions, using models that account for anatomical region, cellular composition, and residual spatial dependence. Testing combinations of choices, as well as changing one choice at a time, reveals interactions between preprocessing, alignment, and annotation.

For each admissible analysis, the association direction, effect size with uncertainty, multiplicity-adjusted significance, and candidate-dependent pathway interpretation were reported. Comparisons across preprocessing variants should use the same biological contrast on comparable effect-size scales across preprocessing variants. Candidate identity may change the pathway interpretation even when the feature-level association is stable; changes in preprocessing or alignment may alter the association itself. A conclusion that persists across these choices supports a regional ion-feature association, whereas a species-specific mechanism remains conditional on resolving chemical identity. Choices that reverse or materially weaken the conclusion should be reported and used to prioritize targeted on-tissue MS/MS, region-matched LC-MS/MS, or independent specimens. Confidence intervals conditional on a single model do not capture all these uncertainties and cannot substitute for chemical confirmation or perturbational evidence.

### Technological bottlenecks: Resolution, sensitivity, and 3-dimensional reconstruction

Spatial resolution, molecular coverage, sensitivity, and acquisition time remain coupled. Routine MALDI-MSI often resolves multicellular regions, although optimized instruments can reach nominal pixel sizes at cellular or submicrometer scales for selected analytes. Nano-secondary-ion MS provides far finer elemental and isotopic imaging but generally does not retain the broad intact-molecule information of MALDI. Transmission-mode MALDI with laser post-ionization has demonstrated a 600-nm nominal pixel size in brain tissue, but this performance depends on specialized optics, sample preparation, and ion statistics [151].

Post-ionization can improve sensitivity at small pixels. In the original MALDI-2 study, a secondary laser increased ion yield by as much as approximately 2 orders of magnitude for some lipid classes; the gain was analyte- and condition-dependent rather than universal [152]. Matrix crystal size, tissue thickness, laser focus, and acquisition geometry also influence effective resolution. Spatial-resolution claims should therefore be reported with raster step, nominal pixel size, sampling footprint, oversampling conditions, molecular coverage, and signal-to-noise criteria.

Pixel dimensions describe the nominal sampling grid, while cell-resolved molecular measurement requires evidence that the recorded ions can be attributed to defined cells. The effective sampling volume depends on the lateral footprint, sampling depth, and section thickness, and can include neighboring cells or extracellular material. Sensitivity and molecular coverage determine which analytes remain detectable at that scale, while delocalization and segmentation errors influence cellular attribution. Linking MSI to ST-defined cell states therefore requires validated cell boundaries, accurate registration, and assignments that account for the MSI sampling footprint. Interpretation should follow the supported attribution scale, from individual cells to cellular mixtures or tissue regions [114].

Three-dimensional MSI usually relies on serial sections. Section loss, nonlinear deformation, variable matrix deposition, and between-run intensity drift can accumulate through a volume, making registration and normalization central parts of the experiment [153]. Sparse acquisition followed by neural reconstruction has reduced sampling burden in proof-of-concept mouse brain and kidney studies, but reconstructed features remain model-dependent and should not replace measured data at critical boundaries [154].

### Clinical translation: Intended use, standardization, and validation

Clinical translation requires a defined intended use before analytical optimization. A discovery map, an adjunct to histopathology, an intraoperative classifier, and a quantitative drug-distribution assay have different requirements for specimen handling, turnaround time, calibration, error tolerance, and operator oversight. Performance must be established on independent specimens representative of the target population, with prespecified quality-control and failure criteria. Analytical feasibility concerns reliable measurement within the workflow; diagnostic performance concerns classification against an appropriate reference standard; clinical utility concerns benefit from using the result for its intended decision. Independent classification accuracy alone does not show improved outcomes. Prospective studies should evaluate decision impact and patient-relevant benefit against current practice, even when the full metabolic mechanism remains unresolved.

FFPE tissue is abundant in pathology archives, but chemical fixation and processing alter molecular recovery. A MALDI Fourier-transform ion-cyclotron-resonance protocol detected extensive molecular information in FFPE tissue and found substantial, but incomplete, overlap with fresh-frozen profiles [155]. This supports archival feasibility, not interchangeability: Analyte-specific recovery, fixation time, storage, antigen retrieval or digestion conditions, and batch effects require validation for each application.

Interlaboratory reproducibility remains a major barrier [156]. A controlled 3-site study of quantitative MSI in a preclinical tissue model showed that agreement is achievable when sample preparation, calibration, acquisition, and analysis are harmonized [157]. That result should not be generalized to all analytes or clinical specimens. Stable-isotope-labeled standards can correct selected sources of variation, but their performance depends on how closely application, extraction, and ionization reproduce those of the endogenous analyte.

Turnaround time is application-specific. A rapid MALDI workflow has demonstrated preparation and acquisition within approximately 30 min in a feasibility setting [158]. Ambient approaches—including DESI, REIMS, and the MasSpec Pen—have also shown near-real-time tissue classification or margin assessment in proof-of-concept studies [35,136,137]. These MS-based tissue-classification technologies require prospective validation, workflow integration, and predefined handling of indeterminate results.

### From molecular cartography to target and drug-distribution studies

Spatial multi-omics can improve target discovery by specifying the cellular and anatomical setting in which a candidate pathway is active. This context helps distinguish a tissue-wide association from a program confined to an invasive front, fibrotic niche, or immune-excluded region. It does not automatically increase predictive validity: Candidate targets still require perturbation, pharmacological tractability, and replication across patients.

Cross-modal maps are most useful when they generate explicit, falsifiable propositions. For example, a transcript-defined enzyme program and a colocated ion pattern with a stated candidate annotation may nominate a pathway for isotope tracing or spatially restricted perturbation. Ligand–receptor analysis can identify candidate communication axes, but it should not be interpreted as measured signaling without protein, metabolite, or functional evidence. Spatial location narrows the hypothesis; it does not complete the causal chain.

Quantitative MSI is especially valuable for tissue pharmacology because it can compare drug abundance with histology and regional molecular responses. In resection specimens from patients with gastrointestinal stromal tumors, MALDI quantitative MSI showed limited imatinib in liver metastases despite higher abundance in surrounding liver and was cross-checked against extract-based LC–MS measurements [36]. The study illustrates how spatial exposure can reveal a potential delivery or retention problem that bulk concentration could mask; the underlying transport mechanism still requires separate testing.

MS-based intraoperative tissue-classification technologies provide a related translational application. DESI-based analysis of brain-tumor specimens [35], REIMS during electrosurgery, and the MasSpec Pen have each demonstrated rapid molecular classification in clinical or preclinical feasibility studies [136,137]. These results support prospective evaluation of margin assessment against standard pathology, with patient-relevant outcomes used to establish clinical benefit. The corresponding standardization and validation requirements for clinical translation are summarized in Table 4.

**Table 4.** Detailed standardization and validation requirements for clinical translation of spatial mass spectrometry. Requirements should be adapted to the analyte class, instrument, specimen, and intended clinical use. Methods should be reported transparently, with numerical acceptance limits validated for the individual assay.

Domain	Primary source of variation or risk	Minimum design and reporting requirements	Quality-control evidence and acceptance criteria	Recommended validation strategy	Maturity and interpretive boundary
Intended use and cohort design	Unclear clinical role, spectrum leakage between training and test data, confounding by site or tissue type, and underpowered subgroup analyses.	Predefine whether the assay is for diagnosis, margin triage, prognosis, or treatment selection. Specify eligibility, reference standard, unit of analysis, specimen flow, exclusions, and handling of indeterminate results.	Prespecified primary endpoint; complete sample accounting; patient-level separation of training, tuning, and test sets; confidence intervals for clinically relevant metrics.	Move from retrospective development to temporally or geographically external validation, then to a prospective study embedded in the intended workflow.	General reporting principles exist, but use-case-specific acceptance limits and regulatory pathways remain incomplete.
Tissue collection and pre-analytical delay	Warm/cold ischemia, delayed freezing or fixation, temperature excursions, blood contamination, freeze–thaw cycles, and variable storage duration can alter labile metabolites and lipids.	Record resection-to-stabilization time, temperature, stabilization method, storage time/temperature, freeze–thaw count, orientation, tissue thickness, and deviations. Use identical handling across comparison groups.	Time-stamped chain of custody; predefined maximum delay; blank and pooled-reference monitoring; exclusion or sensitivity analysis for protocol deviations.	Perform analyte-class-specific stability experiments across realistic delay and storage conditions; verify that classification performance is robust to permitted variation.	Essential but not universally harmonized; acceptance limits must be established for each specimen type and analyte class.
Fresh-frozen versus formalin-fixed, paraffin-embedded tissue	Formalin cross-linking and paraffin processing alter or remove many small molecules and lipids; deparaffinization and antigen/protein retrieval can introduce additional variability.	State fixation time, fixative, block age, deparaffinization, washing/retrieval steps, and whether molecular classes are expected to be preserved. Do not assume equivalence with fresh-frozen tissue.	Matched fresh-frozen/formalin-fixed comparisons where feasible; extraction blanks; monitor representative retained and lost analytes; document batch-specific recovery.	Validate the exact archival workflow for the intended molecular class and classifier; confirm selected targets with orthogonal liquid chromatography–mass spectrometry or immunohistochemistry.	Feasible for selected metabolites, peptides, proteins, and glycans, but preservation is class dependent; claims must remain analyte specific.
Sectioning, sampling, and histopathology	Tissue heterogeneity, section thickness, curling/tearing, tissue microarray core selection, edge effects, and mismatch between the measured and stained section.	Report section thickness, cutting temperature, slide type, section order, sampling scheme, tissue area, missing regions, staining sequence, and pathologist annotation protocol.	Replicate/serial sections; tissue-area thresholds; blinded annotation; agreement assessment for categorical labels; documented registration error.	Quantify within-block and between-block heterogeneity. Test whether a single section/core represents the intended patient-level endpoint.	Routine histology is established, but its integration with molecular pixels remains vulnerable to sampling and annotation bias.
Matrix, solvent, derivatization, and deposition	Matrix chemistry, crystal size, extraction efficiency, spray/sublimation uniformity, humidity, nozzle geometry, and analyte delocalization affect sensitivity and spatial fidelity.	Report matrix/derivatization reagent, solvent and additives, concentration, flow, passes, nozzle height/speed, temperature, humidity, deposition mass or thickness, drying, and time to acquisition.	Coating-uniformity map or gravimetric/optical surrogate; process blanks; replicate coated standards; predefined coefficient-of-variation and delocalization checks.	Use automated deposition where possible and challenge the protocol across days, operators, slide loads, and tissue types. Revalidate after hardware or reagent-lot changes.	Automation can improve throughput, but cross-platform equivalence and universal acceptance criteria are not established.
Instrument setup and acquisition	Mass calibration drift, laser energy/spot size, raster step, ion-source contamination, detector aging, resolving power, polarity, duty cycle, and acquisition-order effects.	Report instrument and software versions, ion source/analyzer, calibration, mass range, resolving power, polarity, raster step, nominal pixel size, sampling footprint, laser/beam settings, scans per pixel, acquisition order, and total time.	Daily system-suitability mixture; lock-mass or recalibration criteria; signal-to-noise, mass-error, resolving-power, and carryover thresholds; randomized or blocked acquisition order.	Assess repeatability, intermediate precision, operator effects, maintenance intervals, and performance after restart. Include realistic tissue controls, not standards alone.	Many parameters are reportable, but platform-specific performance specifications and clinical change-control procedures remain laboratory dependent.
Normalization and ion suppression	Spatially variable salts, matrix, tissue composition, and dominant ions distort relative intensity. Total-ion-current normalization may create artifacts in heterogeneous tissue.	Predefine normalization and preprocessing according to study design; assess global and region-specific changes in signal composition; justify mass exclusions, smoothing, baseline correction, peak picking, and missing-value treatment; retain raw and processed data.	Compare alternative normalization methods; monitor internal or sprayed standards where appropriate; inspect off-tissue signal, dominant-ion regions, and normalization-induced contrast reversal.	Demonstrate robustness across tissue composition and batches; use orthogonal concentration data for key analytes and locked preprocessing for validation cohorts.	No single normalization method is universally valid. Normalized intensity is generally relative and should not be described as concentration without calibration.
Molecular annotation and structural identity	MS1 features can have multiple candidate structures; adducts, isotopologues, in-source fragments, and MALDI matrix ions complicate feature-to-molecule mapping.	Distinguish detected features, formula-level candidates, putative structures, and authentic-standard-supported identifications. Report *m*/*z*, polarity, mass error, adduct/formula, database/version, candidate alternatives, and confidence framework.	Include blanks and off-tissue controls, FDR-controlled formula annotation, and on-tissue MS/MS and authentic standards for key assignments; retain unresolved alternatives.	Use LC-MS/MS or complementary separation/structural methods that discriminate relevant alternatives. Group related ion forms where justified and test pathway/network robustness across alternative assignments.	Formula-level FDR control does not establish isomer identity. Lipid names must match supported structural detail; extract-based validation does not establish the identity contributing to every pixel.
Quantitation and calibration	Ion suppression varies by tissue region; extraction and response are nonlinear; reference standards may not mimic tissue; adjacent-section assays introduce sampling error.	Define whether output is relative, semi-quantitative, or quantitative. Report calibration model/range, weighting, internal standard, tissue-mimetic or dilution design, lower limit, recovery, precision, accuracy, and dilution integrity.	Back-calculated standards and quality controls across the range; spatial homogeneity of internal standard; blank/carryover checks; comparison with validated liquid chromatography–tandem mass spectrometry.	Validate accuracy, precision, selectivity, matrix effects, stability, and robustness in the relevant tissue. Assess multiple analysts, days, instruments, and sites.	Quantitative MSI is feasible for selected analytes, but broad untargeted images should not be interpreted as absolute concentration maps.
Batch effects, reference materials, and multicenter reproducibility	Site, instrument, reagent lot, operator, software, maintenance, and calendar time can dominate biological variation.	Randomize or block specimens, distribute pooled/standardized controls, record lot and maintenance metadata, define batch correction before unblinding, and preserve site labels for sensitivity analysis.	Within-run, between-run, between-operator, and between-site precision; drift plots; predefined failure rules; documented corrective action. Multicenter studies have shown both feasibility and residual site effects.	Conduct ring trials using common specimens and protocols, then test transfer to local workflows. Freeze the analytical pipeline before external validation.	Multicenter proof-of-concept exists; commutable certified tissue reference materials and universal proficiency-testing schemes remain limited.
Multimodal co-registration and 3-dimensional reconstruction	Tissue deformation, serial-section mismatch, optical distortion, different sampling footprints and nominal pixel sizes, missing tissue, and cumulative alignment error can create false colocalization.	Specify whether assays used the same section, serial sections, or separate specimens. Report fiducials, transformation model, interpolation, pixel aggregation, excluded regions, and quantitative registration error.	Landmark/contour error distributions; blinded visual review; negative-control channels; sensitivity analysis to plausible misregistration; section-to-section quality checks.	Validate known anatomical correspondences and repeat analyses at coarser resolution. For 3-dimensional MSI, monitor section order, z-spacing, deformation, and missing-section handling.	Co-registration is methodologically mature in imaging, but biological inference is often more precise than the measured alignment supports.
Data format, metadata, and provenance	Proprietary formats, incomplete metadata, undocumented conversion, software-version changes, and manual region edits impair reproducibility.	Export interoperable imaging data when possible; archive raw files, imzML conversion settings, metadata, annotations, preprocessing code, software/container versions, and audit trail.	File integrity checks; unique identifiers linking spectra, images, specimens, and patients; reproducible rerun from raw data; version-controlled analysis manifest.	Test round-trip conversion on representative files and provide an executable workflow or sufficiently detailed protocol. Retain vendor raw data for adjudication.	imzML is broadly used in research; metadata completeness, feature parity, and long-term clinical data governance remain uneven.
Statistics and machine-learning models	High dimensionality, spatial autocorrelation, pseudoreplication of pixels, multiple testing, class imbalance, center confounding, and data leakage inflate apparent performance.	Use the patient—not the pixel—as the independent unit for splitting and inference. Predefine feature selection, spatial aggregation, multiplicity control, hyperparameter tuning, thresholds, and missing/indeterminate handling.	Nested cross-validation during development; locked external test set; calibration and decision-curve assessment where relevant; subgroup/site performance; confidence intervals obtained at patient level.	Use geographically or temporally distinct cohorts and challenge the model under acquisition shifts. Compare with pathology and simpler baselines; release sufficient code/model detail for audit.	Retrospective classifiers are common, but prospective clinical utility and transportability across sites are less established.
Clinical workflow, governance, and utility	Turnaround time, tissue consumption, biosafety, instrument uptime, operator training, result communication, and unclear action thresholds may prevent routine adoption.	Map the end-to-end workflow; define maximum turnaround time, tissue-preservation requirements, fail-safe pathway, responsible personnel, report format, data retention, and how the result changes care.	Prospective uptime/failure rate, repeat-test rate, indeterminate fraction, concordance with reference pathology, turnaround distribution, and documented clinical actions.	Begin with observational silent-mode deployment, proceed to prospective decision-impact studies, and evaluate patient outcomes or noninferiority only after analytical and diagnostic performance are locked.	Rapid tissue classification is clinically promising, but analytical feasibility should not be equated with improved outcomes or regulatory readiness.

FFPE, formalin-fixed, paraffin-embedded; H&E, hematoxylin and eosin; imzML, imaging mass spectrometry markup language; MSI, mass spectrometry imaging

### Priorities for the next phase

Same-section acquisition shifts the emphasis from aligning neighboring sections to coordinating measurements on a shared specimen. Vicari *et al*. [37] demonstrated a sequential MSI–ST protocol on a single section, and Hendriks *et al*. [58] combined 5-μm MALDI-MSI with Xenium, preserving cell recovery and cell-type assignment despite an approximately 30% reduction in detected transcripts per cell. The next objective is to establish compatible acquisition orders, substrates, and preparation protocols with paired controls that quantify effects on RNA recovery, molecular coverage, and tissue integrity. Using the same tissue plane reduces inter-section correspondence uncertainty but does not eliminate segmentation, sampling-footprint, or assay-compatibility limitations. Optical calibration, fiducial alignment, cell segmentation, and reconciliation of the 2 sampling footprints then become the main spatial tasks.

Spatial metabolite distributions can be linked to nutrient use through *in vivo* stable-isotope labeling followed by ex vivo MSI. Transcript-constrained spatial flux models and isotope-informed MSI provide complementary computational and experimental evidence of pathway activity. Schwaiger-Haber *et al*. [98], for example, combined labeling with spatial isotopologue spectral analysis to estimate regional lipid synthesis and turnover in a mouse glioma model. Future studies can couple such measurements to ST-defined cell states, asking whether a niche with a particular enzyme program also incorporates a labeled substrate into the predicted products. This requires tracer designs that resolve the relevant timescale, measurement of precursor enrichment, correction for natural isotopic abundance, and models that distinguish local synthesis from delivery and exchange. Pairing these estimates with perturbation would connect spatial abundance, pathway use, and functional dependence in a defined tissue niche.

Time-resolved spatial multi-omics at defined stages of development, injury, disease progression, or treatment could reveal how cellular neighborhoods and metabolic programs change together. Because tissue-section MSI consumes material, most such experiments will compare matched specimens or experimental cohorts collected at scheduled times, with repeated biopsy designs where feasible. Temporal models should therefore account for specimen variation, changing cell composition, and anatomical correspondence across time. Pseudotemporal ordering can help organize cell states, while measured time points and pulse–chase labeling establish the timing and duration of metabolic responses. These designs would distinguish persistent niche features from transient responses and expose delays between transcriptional regulation, protein abundance, and metabolic remodeling.

Foundation models may help transfer learned tissue representations across datasets. Nicheformer, trained on dissociated and spatial transcriptomic data, demonstrates that spatial context can be learned and transferred to downstream tasks such as neighborhood-composition prediction [159]. Its use for MSI–ST remains prospective. A useful next step is to combine these transcriptomic priors with representations that preserve MSI mass, adduct, isotope, and annotation-confidence information. We view such models as adaptable priors for cell-state mapping and hypothesis prioritization. Their contribution should be assessed on held-out tissues, patients, and platforms, against simpler task-specific models, with independent tests of molecular assignments and spatial predictions. Predicted metabolite layers require validation against paired MSI measurements.

These directions bring experimental design and computation closer together: Same-section acquisition improves correspondence, tracer and time-resolved studies add dynamic information, and transferable models help interpret larger cohorts. Benchmarking with known correspondences and independent chemical evidence remains the practical foundation for this progress. Instrument studies should report resolution, molecular coverage, sensitivity, and throughput together, and translational programs should retain intended-use definitions, multicenter reproducibility, and prospective validation. The aim is to build reproducible spatial models that explain where metabolic programs operate, how they change, and how they respond to intervention.

## Conclusion

MSI-centered spatial multi-omics provides a route from molecular imaging to tissue-scale mechanistic inference. MSI directly maps the distributions of ion features associated with metabolites, lipids, peptides, proteins, and administered compounds. Annotation and structural validation connect these features to molecular identities, whereas spatial and single-cell sequencing resolves the cellular identities and regulatory programs associated with these molecular patterns. Integrating these measurements localizes molecular patterns, links them to candidate cell populations, and tests the anatomical plausibility of proposed interactions. This framework enables spatially constrained and experimentally testable models of metabolic exchange, inflammatory remodeling, fibrosis, tissue dysfunction, and drug distribution. Kinetic and tracer measurements then characterize flux, turnover, uptake, secretion, and pathway directionality, while functional experiments test the proposed mechanisms.

The reliability of these models ultimately depends on whether the experimental and analytical evidence matches the strength of the biological claim. Registration accuracy defines the spatial scale at which modalities can be linked; unresolved ion identities limit chemical interpretation; and computational colocalization alone cannot establish cellular origin, molecular transport, or causality. Validation should be selected for the intended claim: chemical evidence for molecular identity, independent spatial measurements for attribution, dynamic or perturbational experiments for mechanism, and appropriate cohorts and decision-relevant outcomes for clinical use. These requirements can be addressed in parallel and do not imply that every application must complete the same sequence. Current liver and tumor studies provide direct spatial molecular evidence, whereas the adipose and PE examples primarily supply cellular and anatomical frameworks for future metabolite-resolved investigations. With evidence matched to the claim, spatial multi-omics can progress beyond descriptive molecular cartography toward reproducible explanations of tissue organization and disease mechanisms while also supporting biomarker development, target prioritization, and spatially resolved pharmacological assessment.

Future progress should be judged by how reliably spatial measurements establish molecular identity, cellular context, mechanistic relevance, and suitability for a defined biomedical purpose.

## Data Availability

No new datasets were generated or analyzed in this review.
